# Biological Secondary Metabolites from the *Lumnitzera littorea*-Derived Fungus *Penicillium oxalicum* HLLG-13

**DOI:** 10.3390/md21010022

**Published:** 2022-12-27

**Authors:** Yue Wang, Wenhao Chen, Zhefei Xu, Qiqi Bai, Xueming Zhou, Caijuan Zheng, Meng Bai, Guangying Chen

**Affiliations:** 1 Key Laboratory of Tropical Medicinal Resource Chemistry of Ministry of Education, College of Chemistry and Chemical Engineering, Hainan Normal University, Haikou 571158, China; 2 Key Laboratory of Tropical Medicinal Plant Chemistry of Hainan Province, Haikou 571158, China; 3 Guangxi Key Laboratory of Marine Drugs, Institute of Marine Drugs, Guangxi University of Chinese Medicine, Nanning 530200, China

**Keywords:** *Lumnitzera littorea*, *Penicillium oxalicum*, secondary metabolites, biological activity

## Abstract

Five new compounds, including two cyclopiane diterpenes conidiogenones J and K (**1**–**2**), a steroid andrastin H (**5**), an alkaloid (*Z*)-4-(5-acetoxy-*N*-hydroxy-3-methylpent-2-enamido) butanoate (**6**), and an aliphatic acid (*Z*)-5-acetoxy-3-methylpent-2-enoic acid (**7**), together with ten known compounds (**3**–**4** and **8**–**15**) were isolated from the EtOAc extract of the fermentation broth of the *Lumnitzera littorea*-derived fungus *Penicillium oxalicum* HLLG-13. Their structures were elucidated by 1D, 2D NMR, and HR-ESI-MS spectral analyses. The absolute configurations of **1**, **2**, **5,** and **8** were determined by quantum chemical electronic circular dichroism (ECD) calculations, and the absolute configuration of **8** was determined for the first time. Compound **15** was a new natural product, and its NMR data were reported for the first time. Compounds **5** and **9**–**14** exhibited antibacterial activities against *Staphylococcus epidermidis* and *Candida albicans*, with MIC values ranging from 6.25 to 25 μg/ mL. Compounds **1**–**6** and **9**–**14** showed significant growth inhibition activities against newly hatched *Helicoverpa armigera* Hubner larvae, with IC_50_ values ranging from 50 to 200 μg/mL.

## 1. Introduction

*Lumnitzera littorea* (Jack) Voigt is a mangrove tree that has been included on the list of national key protected wild plants (the first batch) (Level II) approved by the State Council of China on 4 August 1999. According to the literature reports, different types of active compounds from *Lumnitzera* have been isolated, such as hepatoprotective flavonoids and phenolic glycosides [1], antileishmanial macrolides [2], cytotoxic polyketones [3], and anti-angiogenic and anti-inflammatory neolignans [4]. Due to the shortage of *L. littorea*, the study of bioactive secondary metabolites from the *L. littorea*-derived endophytic fungus is necessary. Only four articles about the secondary metabolites from the endophytic fungi of *Lumnitzera* have been reported [5,6,7,8], including an article about antibacterial terpenoids [5], one about cytotoxic polyketones [6], another about cytotoxic oxygenated meroterpenoids [7], and one about steroids with α-glucosidase inhibitory activity [8].

**Figure 1:**
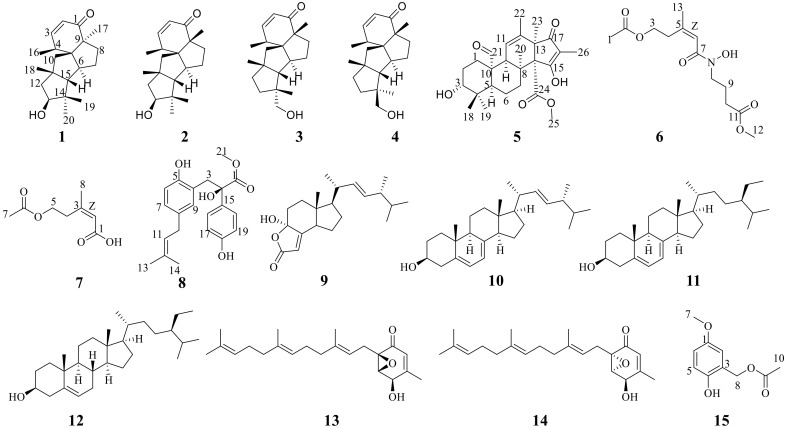
The structures of compounds **1**–**15**.

During our exploration of the structurally diverse and bioactive compounds from mangrove-derived fungi, some new bioactive compounds have been found [9,10,11,12]. In the previous study, cytotoxic oxygenated meroterpenoids and steroids with α-glucosidase inhibitory activity had been isolated from the secondary metabolites of two endophytic fungi: *Penicillium* sp. HLLG-122 and *Penicillium sclerotiorum* HLL113, which were both isolated from the roots of the *L. littorea*. [7,8]. In our continuing research, the endophytic fungus *Penicillium oxalicum* HLLG-13, obtained from the roots of *L. littorea* and collected from the Tielugang Mangrove Reserve in Sanya, was selected for further research because its EtOAc extract showed antibacterial activity and growth inhibition activity against newly hatched *H. armigera* Hubner larvae. Five new compounds (**1**–**2** and **5**–**7**) and ten known compounds (**3**–**4** and **8**–**15**) (Figure 1) were isolated from the EtOAc extract of the fermentation broth from *P. oxalicum* HLLG-13. In this study, we report the isolation, structure elucidation, antibacterial activity, and growth inhibition activity of these compounds against newly hatched *H. armigera* Hubner larvae.

## 2. Results and Discussion

**Figure 2:**
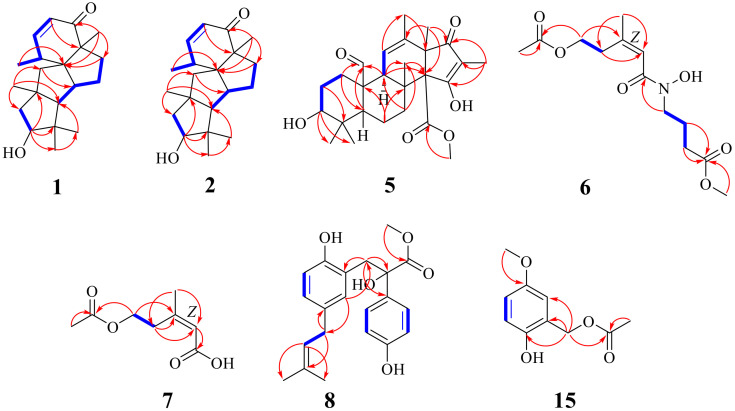
^1^H-^1^H COSY and key HMBC correlations for **1**–**2**, **5**–**8**, and **15**.

Compound **1** was isolated as a brownish yellow oil. Its molecular formula was established by HR-ESI-MS (*m/z* 325.2135 [M + Na]^+^, calcd. for 325.2143) to be C_20_H_30_O_2_ with six degrees of unsaturation (Supplementary Materials). ^1^H NMR data (Table 1) showed two olefinic proton signals at *δ*_H_ 5.94 (1H, dd, *J* = 10.0, 1.2 Hz, H-2) and 7.08 (1H, dd, *J* = 10.0, 5.6 Hz, H-3); five methyl signals at *δ*_H_ 1.26 (3H, d, *J* = 7.3 Hz, H-16), 1.20 (3H, s, H-17), 1.12 (3H, s, H-18), 1.12 (3H, s, H-19), and 0.98 (3H, s, H-20); four methine signals at *δ*_H_ 2.79 (1H, m, H-4), 2.41 (1H, m, H-6), 4.08 (1H, dd, *J* = 10.4, 7.2 Hz, H-13), and 1.64 (1H, m, H-15); and six methylene signals at *δ*_H_ 1.63 (1H, m, H-7a), 1.23 (1H, m, H-7b), 2.06 (2H, m, H-8), 1.74 (2H, m, H-10), 2.18 (1H, d, *J* = 14.8 Hz, H-12a), and 1.49 (1H, d, *J* = 14.8 Hz, H-12b). The ^13^C NMR data (Table 2) combined with the DEPT spectrum exhibited twenty carbon resonances, including one carbonyl carbon at *δ*_C_ 208.0 (C-1); two olefinic carbons at *δ*_C_ 128.0 (C-2) and 157.1 (C-3); five methyl carbons at *δ*_C_ 18.8 (C-16), 21.5 (C-17), 35.1 (C-18), 23.1 (C-19), and 29.5 (C-20); four methylene carbons at *δ*_C_ 35.6 (C-7), 40.0 (C-8), 50.1 (C-10), and 43.5 (C-12); four methine carbons at *δ*_C_ 40.2 (C-4), 55.8 (C-6), 78.5 (C-13), and 74.0 (C-15); and four quaternary carbons at *δ*_C_ 61.9 (C-5), 58.7 (C-9), 36.6 (C-11), and 57.4 (C-14). These signals were closely related to those of **3** [13], except for the presence of one hydroxyl-methylene group at *δ*_H_ 4.08 (1H, dd, *J* = 10.4, 7.2 Hz, H-13) and *δ*_C_ 78.5 (C-13), and one methyl signal at *δ*_H_ 1.12 (3H, s, H-20) and *δ*_C_ 29.5 (C-20) in **1.** The absence of one hydroxymethyl group at *δ*_H_ 3.32 (3H, d, *J* = 10.9 Hz) and *δ*_C_ 72.0 in **3** indicated that the methylene at C-13 in **3** was replaced by a hydroxyl-methylene group in **1**, and the hydroxymethyl group at C-20 in **3** was replaced by a methyl group in **1**. These results were further confirmed by the ^1^H-^1^H COSY and HMBC spectra. The ^1^H-^1^H COSY correlation between H-12 to H-13, combined with the HMBC correlations from H-12 to C-13, H-13 to C-19, H-15 to C-19, H-19 to C-15/C-20, and H-20 to C-13/C-15/C-19, confirmed the structure (Figure 2). Hereto, the planar structure of **1** was elucidated.

**Figure 3:**
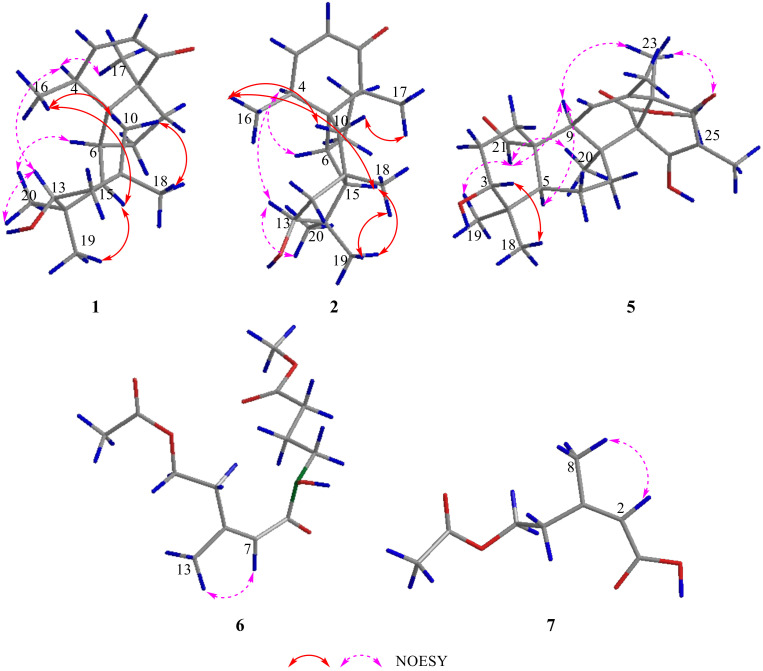
Key NOESY correlations for **1**–**2** and **5**–**7**.

The relative configuration of **1** was elucidated by the NOESY correlations (Figure 3). The NOE relationships of H-4 to H-13/Me-17 and Me-20 to H-6/H-13 indicated that H-4, H-6, H-13, Me-17, and Me-20 were in *α*-orientation. The relationships of H-15 to Me-16/Me-19 and H-10 to H-16/Me-18 indicated that H-10, H-15, Me-16, Me-18, and Me-19 were in *β*-configuration. Thus, the relative configuration of **1** was determined to be 4R*, 5R*, 6R*, 9R*, 11R*, 13R*, and 15R*.

**Figure 4:**
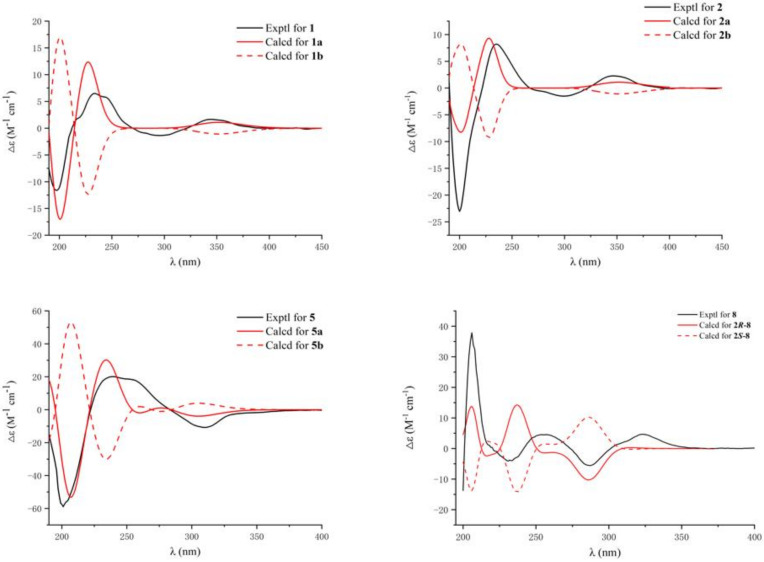
Experimental CD spectra and the calculated ECD spectra of compounds **1**–**2**, **5**, and **8**.

In order to determine the absolute configuration of **1**, the theoretical electronic circular dichroism (ECD) spectra of two possible stereoisomers of (4*R*, 5*R*, 6*R*, 9*R*, 11*R*, 13*R*, 15*R*)-**1** (**1a**) and its enantiomer (**1b**) were calculated using time-dependent density-functional theory (TDDFT) calculation, and the calculated ECD curve of isomer **1a** revealed good agreement with the experimental one (Figure 4). Therefore, the absolute configuration of **1** were assigned as 4*R*, 5*R*, 6*R*, 9*R*, 11*R*, 13*R*, and 15*R* and named as conidiogenone J.

Compound **2** was isolated as a brownish yellow oil. HR-ESI-MS, the ^1^H NMR, and ^13^C NMR data (Table 1 and Table 2) showed that **2** was almost the same as **1**; thus, compound **2** has the same planar structure as **1**. The relative configuration of **2** was determined by the NOESY correlations (Figure 3). The NOE relationships of H-4 to H-6/H-13 and H-13 to Me-20 indicated that H-4, H-6, H-13, and Me-20 were in *α*-orientation. The relationships of H-15 to Me-16/Me-19, H-10 to Me-16/Me-17, and Me-18 to Me-19 indicated that H-10, H-15, Me-16, Me-17, Me-18, and Me-19 were in *β*-configuration. Thus, the relative configuration of **2** was determined to be 4R*, 5R*, 6R*, 9S*, 11R*, 13R*, and 15R*. The calculated ECD spectrum of (4*R*, 5*R*, 6*R*, 9*S*, 11*R*, 13*R*, 15*R*)-**2** (**2a**) showed good agreement with the experimental spectrum of **2** (Figure 4). Therefore, the absolute configuration of **2** were assigned as **2a** and named as conidiogenone K.

Compound **5** was obtained as a brownish yellow oil, and it was determined to have the molecular formula C_26_H_36_O_6_ on the basis of positive HR-ESI-MS (*m/z* 467.2404 [M + Na]^+^, calcd. for 467.2410) with nine degrees of unsaturation. ^1^H NMR spectrum (Table 1) of **5** revealed the presence of one singlet olefinic proton signal at *δ*_H_ 5.21 (1H, s, H-11); one aldehyde proton signal at *δ*_H_ 10.07 (1H, s, H-21); one methoxy signal at *δ*_H_ 3.49 (3H, s, H-25); six methyl signals at *δ*_H_ 0.89 (3H, s, H-18), 0.67 (3H, s, H-19), 1.11 (3H, s, H-20), 1.65 (3H, s, H-22), 1.02 (3H, s, H-23), and 1.50 (3H, s, H-26); three methine signals at *δ*_H_ 3.17 (1H, m, H-3), 1.76 (1H, m, H-5), and 2.00 (1H, q, *J* = 2.7 Hz, H-9); and eight methylene signals at *δ*_H_ 2.07 (1H, t, *J* = 13.3 Hz, H-1a), 1.36 (1H, t, *J* = 13.3 Hz, H-1b), 1.36 (1H, m, H-2a), 1.02 (1H, m, H-2b), 2.00 (1H, m, H-6a), 1.53 (1H, d, *J* = 13.2 Hz, H-6b), 2.93 (1H, t, *J* = 13.2 Hz, H-7a), and 2.06 (1H, t, *J* = 13.2 Hz, H-7b). The ^13^C NMR data (Table 2), combined with the DEPT spectrum of **5,** revealed the presence of twenty-six carbon, including one aldehyde carbon at *δ*_C_ 206.0 (C-21); one ester carbon at *δ*_C_ 170.8 (C-24); one carbonyl carbon at *δ*_C_ 195.5 (C-17); one oxygenated olefinic carbon at *δ*_C_ 186.2 (C-15); three olefinic carbons at *δ*_C_ 122.0 (C-11), 135.5 (C-12), and 111.5 (C-16); one oxomethyl carbon at *δ*_C_ 51.6 (C-25); six methyl carbons at *δ*_C_ 27.4 (C-18), 21.3 (C-19), 19.1 (C-20), 19.7 (C-22), 15.5 (C-23), and 6.9 (C-26); four methylene carbons at *δ*_C_ 27.0 (C-1), 25.8 (C-2), 16.5 (C-6), and 32.2 (C-7); three methine carbons at *δ*_C_ 73.5 (C-3), 46.4 (C-5), and 53.1 (C-9); and five quaternary carbons at *δ*_C_ 37.4 (C-4), 41.2 (C-8), 51.7 (C-10), 55.7 (C-13), and 66.9 (C-14). The above data showed that the planar structure of **5** was similar to that of andrastin E [14]. The obvious differences were the appearance of one aldehyde signal at *δ*_H_ 10.07 (1H, s) for H-21, instead of one methyl signal at *δ*_H_ 0.90 (3H, s) in the ^1^H NMR spectrum. Furthermore, the ^13^C NMR data from one aldehyde carbon at *δ*_C_ 206.0 (CH) for C-21 were observed, instead of one methyl carbon at *δ*_C_ 16.7 (CH_3_) in andrastin E, indicating the methyl of andrastin E was replaced by an aldehyde group in **5**. The HMBC correlations (Figure 2) from H-2 to C-21, H-5 to C-21, H-9 to C-21, and H-21 to C-1/C-10 verified the statement above. Hereto, the planar structure of **5** was elucidated.

The relative configuration of compound **5** was determined by the NOESY correlations (Figure 3). The NOE relationships of H-21 to H-19/H-9/H-20, H-9 to H-5/H-23, and Me-25 to Me-23 indicated that H-5, H-9, Me-19, Me-20, H-21, Me-23, and Me-25 were in *α*-orientation. The relationships of H-3 to H-18 indicated that H-3 and Me-18 were in *β*-configuration. Thus, the relative configurations of **5** were determined to be 3R*, 5S*, 8S*, 9S*, 10S*, 13R*, and 14R*.

The absolute configuration was assigned by the experimental and calculated ECD spectra. The ECD spectrum of (3*R*, 5*S*, 8*S*, 9*S*, 10*S*, 13*R*, 14*R*)-**5** (**5a**) and its enantiomer (**5b**) were calculated using TDDFT in MeOH. As shown in Figure 4, the calculated spectrum of **5a** agreed well with the experimental spectrum. Therefore, the absolute configuration of **5** was assigned as **5a** and named as andrastin H.

Compound **6** was obtained as a brownish yellow oil, and its molecular formula was determined to be C_13_H_21_NO_6_ on the basis of negative HR-ESI-MS (*m/z* 286.1290 [M − H]^−^, calcd. for 286.1291), indicating four degrees of unsaturation. ^1^H NMR data (Table 1) displayed one olefinic hydrogen signal at *δ*_H_ 6.30 (1H, br s, H-6); five methylene signals at *δ*_H_ 4.11 (2H, t, *J* = 7.2 Hz, H-3), 3.53 (2H, t, *J* = 6.8 Hz, H-8), 2.79 (2H, m, H-4), 2.30 (2H, t, *J* = 7.6 Hz, H-10), and 1.77 (2H, m, H-9); and three methyl signals at *δ*_H_ 3.58 (3H, s, H-12), 1.98 (3H, s, H-1), and 1.87 (3H, br s, H-13). A total of thirteen carbon signals (including three carbonyl carbons at *δ*_C_ 170.2 (C-2), 166.2 (C-7), and 173.0 (C-11); two olefinic carbons at *δ*_C_ 149.5 (C-5) and 117.6 (C-6); five methylene carbons at *δ*_C_ 62.4 (C-3), 31.9 (C-4), 46.2 (C-8), 21.8 (C-9), and 30.4 (C-10); one methoxy group at *δ*_C_ 51.2 (C-12); and two methyl groups at *δ*_C_ 20.7 (C-1) and 25.2 (C-13)) were exhibited in the ^13^C NMR data (Table 2), combined with the DEPT spectrum. The ^1^H-^1^H COSY correlations from H-3 to H-4, H-8 to H-9, and H-9 to H-10, combined with the HMBC correlations (Figure 2) from H-1 to C-2, H-3 to C-2/C-5, H-4 to C-6/C-13, H-8 to C-7/C-10, H-9 to C-11, H-10 to C-8, H-12 to C-11, and H-13 to C-4/C-6. Therefore, the planar structure of **6** was elucidated as showed in Figure 1. The *Z*-configuration of the double bond was determined from the correlation of H-6 to Me-13 in the NOESY spectrum (Figure 3). Thus, the structure of **6** was established and named as methyl (*Z*)-4-(5-acetoxy-*N*-hydroxy-3-methylpent-2-enamido) butanoate.

Compound **7** was isolated as a yellow oil with the molecular formula C_8_H_12_O_4_ (three degrees of unsaturation) by the HR-ESI-MS spectrum (*m/z* 173.0807 [M + H]^+^, calcd. for 173.0814). An analysis of the ^1^H NMR data (Table 1) indicated that **7** has an olefinic hydrogen signal at *δ*_H_ 5.77 (1H, br s, H-2), two methylene signals at *δ*_H_ 4.21 (2H, t, *J* = 6.8 Hz, H-5) and 2.95 (2H, t, *J* = 6.8 Hz, H-4), and two methyl signals at *δ*_H_ 2.01 (3H, s, H-7) and 1.95 (3H, br s, H-8). The ^13^C NMR data (Table 2), combined with the DEPT spectrum, exhibited eight carbon resonances, including two carbonyl carbons at *δ*_C_ 169.4 (C-1) and 172.7 (C-6), two olefinic carbons at *δ*_C_ 119.5 (C-2) and 157.1 (C-3), two methylene carbons at *δ*_C_ 33.3 (C-4) and 63.8 (C-5), and two methyl groups at *δ*_C_ 20.8 (C-7) and 25.7 (C-8). The ^1^H-^1^H COSY correlation from H-4 to H-5, combined with the HMBC correlations (Figure 2) from H-2 to C-4/C-8, H-4 to C-2/C-8, H-5 to C-3/C-6, H-7 to C-6, and H-8 to C-2. On the basis of these results, the planar structure of **7** was elucidated. The *Z*-configuration of the double bond was determined from the correlation of H-2 to Me-8 in the NOESY spectrum (Figure 3). Thus, the structure of **7** was established and named as (*Z*)-5-acetoxy-3-methylpent-2-enoic acid.

Compound **8** was isolated as a yellow amorphous powder. Its molecular formula was established by HR-ESI-MS (*m/z* 379.1529 [M + Na]^+^, calcd. for 379.1521) to be C_21_H_24_O_5_ with ten degrees of unsaturation. Compared with that of the literature [15], the 1D NMR data (Table 1 and Table 2) of **8** closely resembled those of stocksiloate, which was isolated from *Vincetoxicum stocksii*, and the absolute configuration remained to be determined due to certain limitations. In order to determine the absolute configuration of **8**, the theoretical ECD spectra of two possible stereoisomers of 2*R* and 2*S* were calculated using TDDFT calculation, and the calculated ECD curve of isomer 2*R* revealed a good agreement with the experimental one (Figure 4). Therefore, the absolute configuration of **8** was assigned as 2*R*-form and named as methyl 2*R*-stocksiloate.

Compound **15** was isolated as a white amorphous powder, and its molecular formula was established as C_10_H_12_O_4_ by HR-ESI-MS (*m/z* 195.0660 [M − H]^−^, calcd. for 195.0657) with five degrees of unsaturation. The ^1^H NMR (Table 1) showed three aromatic hydrogen signals at *δ*_H_ 6.75 (1H, d, *J* = 3.2 Hz, H-2), 6.80 (1H, d, *J* = 8.8 Hz, H-5), and 6.70 (1H, dd, *J* = 3.2, 8.8 Hz, H-6); one methylene signal at *δ*_H_ 5.06 (2H, s, H-8); and two methyl signals at *δ*_H_ 3.77 (3H, s, H-7) and 2.07 (3H, s, H-10). The ^13^C NMR data (Table 2) combined with the DEPT spectrum exhibited ten carbon resonances, including a carbonyl carbon at *δ*_C_ 172.8 (C-9); a benzene moiety at *δ*_C_ 152.2 (C-1), 117.3 (C-2), 126.5 (C-3), 152.1 (C-4), 113.1 (C-5), and 116.3 (C-6); a methoxy carbon at *δ*_C_ 56.6 (C-7); a methylene carbon at *δ*_C_ 62.7 (C-8); and a methyl carbon at *δ*_C_ 20.8 (C-10). The ^1^H-^1^H COSY correlation from H-5 to H-6 was combined with the HMBC correlations from H-2 to C-4/C-6/C-8, H-5 to C-1/C-3, H-6 to C-2/C-4, H-8 to C-2/C-3/C-4/C-9, H-7 to C-1, and H-10 to C-9 (Figure 2). Thus, compound **15** was identified as (2-hydroxy-5-methoxyphenyl) methyl acetate. According to the available literature, compound **15** was obtained as the intermediate in the synthesis reaction [16,17,18,19]; thus, it was a new natural product reported here for the first time, and its NMR data were reported for the first time.

By comparing physical and spectroscopic data with literatures, the eight known compounds were identified as conidiogenone D (**3**) [13], conidiogenone C (**4**) [13], demethylincisterol A3 (**9**) [20], ergosterol (**10**) [21], *Δ*^7^-sitosterol (**11**) [22], (−)-*β*-sitosterol (**12**) [23], 7-deacetoxyyanuthone A (**13**) [24], and (1*S*,5*R*,6*S*)-5-Hydroxy-4-methyl-1-[(2*E*,6*E*)-3,7,11-trimethyl-2,6,10-dodecatrien-1-yl]-7-oxabicyclo[4.1.0]hept-3-en-2-one (**14**) [25]. Compounds **1**–**4** were cyclopiane diterpenes with a unique 6/5/5/5 tetracyclic skeleton, and this type of compound is very rarely found from a natural source. Only thirteen compounds have been found in the existing literature, and most of them were mainly isolated as secondary metabolites from the genus of *Penicillum* [13,26,27,28,29,30].

The antibacterial activities of all compounds were determined against eight pathogenic bacteria (*S. aureus* (ATCC 25923), *E. coli* (ATCC 25922), *C. albicans* (ATCC 14053), *S. epidermidis* (ATCC 49134), *P. aeruginosa* (ATCC 17749), *V. harveyi* (ATCC 25919), *V. alginolyticus* (ATCC 33787), and *V. parahaemolyticus* (ATCC 27969)) by the microplate assay method [31]. Compounds **5** and **9**–**14** exhibited obvious antibacterial activities against *S. epidermidis* and *C. albicans*, with the MIC values ranging from 6.25 to 25 μg/ mL (Table 3).

The growth inhibition activities against newly hatched *H. armigera* Hubner larvae were tested using the assay described by Guo [32]. Compound **5** exhibited obvious insecticidal activity against newly hatched *H. armigera* Hubner larvae, with an IC_50_ value of 50 μg/mL, which was equivalent to that of the positive control (azadirachtin); and compounds **1**–**4**, **6**, and **9**–**14** also showed growth inhibition activities against newly hatched *H. armigera* Hubner larvae, with IC_50_ values ranging from 100 to 200 μg/ mL (Table 4).

## 3. Materials and Methods

### 3.1. General Experimental Procedures

Optical rotations were measured on an Anton paar MCP 5100 modular circular polarimeter (JASCO, Tokyo, Japan). ECD spectra were recorded on a Boilogic Mos-500 spectrometer (JASCO, Tokyo, Japan). IR spectra were recorded on a Nicolet 6700 spectrophotometer (Thermo Scientific, Madison, WI, USA). UV spectra were recorded on a Beckman DU 640 spectrophotometer (JASCO, Tokyo, Japan). The 1D and 2D NMR spectra were obtained with a Bruker AV spectrometer (400 MH_Z_ for ^1^H and 100 MH_Z_ for ^13^C, (Bruker Corporation, Basel, Switzerland) or a JNM-ECZS spectrometer (600 HM_Z_ for ^1^H and 125 MH_Z_ for ^13^C, (JEOL, Tokyo, Japan), using Methanol-*d*_4_ or DMSO-*d*_6_ as a solvent. TMS was used as an internal standard. HR-ESI-MS spectra were measured on a Bruker APEX II spectrometer (Billerica, MA, USA). Silica gel (Qing Dao Hai Yang Chemical Group Co., Qingdao, China; 200–300 mesh) and octadecylsilyl silica gel (YMC; 12 μm–50 μm) were used for column chromatography (CC). Precoated silica gel plates (Yan Tai Zi Fu Chemical Group Co., Yan Tai, China; G60, F-254) were used for thin layer chromatography (TLC). Semi-preparative HPLC was performed on an Agilent 1260 LC series with a DAD detector using an Agilent Eclipse XDB-C_18_ column (250 × 9.4 mm, 5 μm, Agilent Corporation, Santa Clara, CA, USA).

### 3.2. Fungal Materials

The fungus HLLG-13 was isolated from the roots of the mangrove *L. littorea* (Jack) Voigt. The *L. littorea* was collected in Tielugang, Sanya city, Hainan province, in November 2018 and identified by Yukai Chen, associate professor of the College of Life Sciences, Hainan Normal University. This strain was deposited in the Key Laboratory of Tropical Medicinal Resource Chemistry of Ministry of Education, College of Chemistry and Chemical Engineering, Hainan Normal University, Haikou, Hainan, China. The fungus was identified according to its morphological traits and a molecular protocol by amplification and sequencing of the DNA of the ITS region of the rRNA gene. Its base pair ITS sequence had 99% sequence identity to that of *P. oxalicum*. Therefore, the fungal strain was identified as *P. oxalicum*. The sequence data have been submitted to GenBank, with an accession number of OK560165. 

### 3.3. Fermentation, Extraction, and Isolation

The seed culture was prepared in a potato liquid medium (30 g sea salt in 1 L of potato infusion in 1 L × 4 Erlenmeyer flasks, each containing 300 mL seed medium), and incubated on a rotary shaker (160 rpm) for 3 days at 28 °C. In total, 50 mL seed culture was then transferred into 1 L Erlenmeyer flasks with a solid rice medium, for a total of 200 bottles of fermentation (each flask contained 80 g rice, 3.0 g sea salt, and 100 mL water) at 28 °C for 28 days. The medium was extracted repeatedly with EtOAc to obtain the corresponding extracts.

The EtOAc extracts were concentrated in vacuo to yield an oily residue (61.27 g). The total crude extract was subjected to silica gel column chromatography (CC) eluted with petroleum ether/EtOAc (*v/v*, gradient 100:0–0:100) and EtOAc/CH_3_OH (*v/v*, gradient 100:0–0:100) to generate nine fractions (Fr.1–Fr.9). Fr.2 (12.7 g) was fractionated by silica gel CC eluted with petroleum ether/EtOAc (*v/v*, gradient 100:0–0:100) to obtain six fractions (Fr.2-1–Fr.2-6) by the TLC analysis. We obtained **11** (9.43 mg) by crystallization from Fr2-1. Fr.3 (4.6 g) was fractionated by silica gel CC eluted with petroleum ether/EtOAc (*v/v*, gradient 100:0–0:100) to obtain five fractions (Fr.3-1–Fr.3-5) by the TLC analysis. We obtained **10** (12.32 mg) by crystallization from Fr.3-1, and **12** (8.25 mg) was obtained by crystallization from Fr.3-3. Fr.4 (3.2 g) was fractionated by reverse phase silica gel using a gradient elution of CH_3_OH/H_2_O system (1:9-10:0) to obtain thirteen fractions (Fr.4-1–Fr.4-13) by the TLC analysis. Fr.4-1 (722.8 mg) was further purified by semi-preparative HPLC using Agilent Eclipse XDB-C_18_ (250 × 9.4 mm, 5 μm) with CH_3_CN/H_2_O (32:68, *v*/*v*) to obtain four fractions (Fr.4-1-1–Fr.4-1-4). Fr.4-1-1 (206.3 mg) was further purified by semi-preparative HPLC using Agilent Eclipse XDB-C_18_ (250 × 9.4 mm, 5 μm) with CH_3_OH/H_2_O (38:62, *v*/*v*) to obtain **7** (37.62 mg). Fr.4-1-3 (43.2 mg) was further purified by semi-preparative HPLC using Agilent Eclipse XDB-C_18_ (250 × 9.4 mm, 5 μm) with CH_3_CN/H_2_O (22:78, *v*/*v*) to obtain **15** (3.99 mg). Fr.4-5 (348.2 mg) was further purified by semi-preparative HPLC using Agilent Eclipse XDB-C_18_ (250 × 9.4 mm, 5 μm) with CH_3_OH/H_2_O (62:38, *v*/*v*) to obtain **1** (12.49 mg), **2** (10.6 mg), and **3** (41.04 mg). Fr.4-7 (106.3 mg) was further purified by semi-preparative HPLC using Agilent Eclipse XDB-C_18_ (250 × 9.4 mm, 5 μm) with CH_3_CN/H_2_O (48:52, *v*/*v*) to obtain **4** (11.52 mg). Fr.4-11 (405.7 mg) was further purified by semi-preparative HPLC using Agilent Eclipse XDB-C_18_ (250 × 9.4 mm, 5 μm) with CH_3_CN/H_2_O (72:28, *v*/*v*) to obtain **9** (9.57 mg), **13** (140.49 mg), and **14** (23.18 mg). Fr.5 (4.1 g) was fractionated by reverse phase silica gel using a gradient elution of CH_3_OH/H_2_O system (1:9–10:0) to obtain nine fractions (Fr.5-1–Fr.5-9) by the TLC analysis. Fr.5-6 (124.8 mg) was further purified by semi-preparative HPLC using Agilent Eclipse XDB-C_18_ (250 × 9.4 mm, 5 μm) with CH_3_CN/H_2_O (46:54, *v*/*v*) to obtain **8** (20.09 mg). Fr.6 (3.9 g) was fractionated by reverse phase silica gel using a gradient elution of CH_3_OH/H_2_O system (1:9–10:0) to obtain fifteen fractions (Fr.6-1–Fr.6-15) by the TLC analysis. Fr.6-4 (77.6 mg) was further purified by semi-preparative HPLC using Agilent Eclipse XDB-C_18_ (250 × 9.4 mm, 5 μm) with CH_3_CN/H_2_O (19:81, *v*/*v*) to obtain **6** (7.23 mg). Fr.6-9 (802.5 mg) was further purified by semi-preparative HPLC using Agilent Eclipse XDB-C_18_ (250 × 9.4 mm, 5 μm) with CH_3_CN/H_2_O (2%CH_3_COOH) (32:64, *v*/*v*) to obtain **5** (198.0 mg).

*Conidiogenone J* (**1**): brownish yellow oil; [*α*]^25^_D_ +13.8 (*c* 0.10, MeOH); UV (MeOH) *λ*_max_ (log *ε*) 261 (0.56); IR (KBr) *ν*_max_ 3426, 2952, 2865, 1661, 1453, 1386, 1272, 1073, 1024 cm^−1^; CD (*c* 0.0013, MeOH) *λ*_max_ (*Δε*) 197 (−11.56), 232 (+6.38), 295 (−1.35), 344 (+1.76) nm; ^1^H and ^13^C NMR data, see Table 1 and Table 2; HR-ESI-MS *m/z*: 325.2135 [M + Na]^+^ (C_20_H_30_O_2_Na, calcd. for 325.2143).

*Conidiogenone K* (**2**): brownish yellow oil; [*α*]^25^_D_ +8.6 (*c* 0.10, MeOH); UV (MeOH) *λ*_max_ (log *ε*) 261 (0.52); IR (KBr) *ν*_max_ 3423, 2956, 2863, 1657, 1446, 1382, 1275, 1068, 1020 cm^−1^; CD (*c* 0.002, MeOH) *λ*_max_ (*Δε*) 200 (−23.01), 235 (+8.12), 299 (−1.52), 346 (+2.30) nm; ^1^H and ^13^C NMR data, see Table 1 and Table 2; HR-ESI-MS *m/z*: 325.2134 [M + Na]^+^ (C_20_H_30_O_2_Na, calcd. for 325.2143).

*Andrastin H* (**5**): brownish yellow oil; [*α*]^25^_D_ +67.6 (*c* 0.10, MeOH); UV (MeOH) *λ*_max_ (log *ε*) 205 (2.46), 242 (1.82); IR (KBr) *ν*_max_ 3442, 2955, 2886, 1744, 1692, 1611, 1463, 1382, 1328, 1215, 1147, 1020, 1007 cm^−1^; CD (*c* 0.002, MeOH) *λ*_max_ (*Δε*) 201 (−58.01), 238 (+19.65), 3.9 (−10.99) nm; ^1^H and ^13^C NMR data, see Table 1 and Table 2; HR-ESI-MS *m/z*: 467.2404 [M + Na]^+^ (C_26_H_36_O_6_Na, calcd. for 467.2410).

*(Z)-4-(5-acetoxy-N-hydroxy-3-methylpent-2-enamido) butanoate* (**6**): brownish yellow oil; [*α*]^25^_D_ +23.8 (*c* 0.10, MeOH); UV (MeOH) *λ*_max_ (log *ε*) 219 (2.38); IR (KBr) *ν*_max_ 3415, 2929, 1735, 1618 cm^−1^; ^1^H and ^13^C NMR data, see Table 1 and Table 2; HR-ESI-MS *m/z*: 286.1290 [M − H]^−^ (C_13_H_20_NO_6_, calcd. for 286.1291).

*(Z)-5-acetoxy-3-methylpent-2-enoic acid* (**7**): yellow oil; [*α*]^25^_D_ +14.2 (*c* 0.10, MeOH); UV (MeOH) *λ*_max_ (log *ε*) 215 (1.37); IR (KBr) *ν*_max_ 3473, 3414, 1639, 1243 cm^−1^; ^1^H and ^13^C NMR data, see Table 1 and Table 2; HR-ESI-MS *m/z*: 173.0807 [M + H]^+^, (C_8_H_13_O_4_, calcd. for 173.0814).

*2R-stocksiloate* (**8**): yellow amorphous powder; m.p. 162–163 °C; [*α*]^25^_D_ +68.4 (*c* 0.10, MeOH); UV (MeOH) *λ*_max_ (log *ε*) 206 (1.48), 306 (0.95); IR (KBr) *ν*_max_ 3419, 1740, 1613, 1514, 1265 cm^−1^; CD (*c* 0.0015, MeOH) *λ*_max_ (*Δε*) 206 (+37.52), 232 (−4.10), 256 (+4.52), 286 (−5.62), 323 (+4.66) nm; ^1^H and ^13^C NMR data, see Table 1 and Table 2; HR-ESI-MS *m/z*: 379.1529 [M + Na]^+^ (C_21_H_24_O_5_Na, calcd. for 379.1521).

*(2-hydroxy-5-methoxyphenyl) methyl acetate* (**15**): white amorphous powder; m.p. 126–127 °C; [*α*]^25^_D_ +12.6 (*c* 0.10, MeOH); UV (MeOH) *λ*_max_ (log *ε*) 208 (1.85), 226 (1.95), 295 (1.07); IR (KBr) *ν*_max_ 3534, 3442, 1697, 1264 cm^−1^; ^1^H and ^13^C NMR data, see Table 1 and Table 2; HR-ESI-MS *m/z*: 195.0660 [M − H]^−^ (C_10_H_11_O_4_, calcd. for 195.0657).

### 3.4. Biological Assays

#### 3.4.1. Antibacterial Activity

The antibacterial activities of all compounds against eight pathogenic bacteria (*S. aureus* (ATCC 25923), *E. coli* (ATCC 25922), *C. albicans* (ATCC 14053), *S. epidermidis* (ATCC 49134), *P. aeruginosa* (ATCC 17749), *V. harveyi* (ATCC 25919), *V. alginolyticus* (ATCC 33787), and *V. parahaemolyticus* (ATCC 27969)) were determined by the microplate assay method. The activated pathogenic bacteria were inoculated into the nutrient broth medium. The concentration of the test group and positive control was 1 mg/mL. The antibacterial effect was evaluated by full wavelength multifunctional microplate reader measurement at 630 nm; the broth medium containing pathogenic bacteria was used as the blank group and DMSO as the negative control; and ciprofloxacin was used as the positive control. 

#### 3.4.2. Growth Inhibition Activities against Newly Hatched *H. armigera* Hubner Larvae

In the test, there were three groups, each containing three neonate larvae of *H. armigera* Hubner, and the tested compounds were dissolved in DMSO at a concentration of 1 mg/mL. The insecticidal activity was investigated by adding the serial dilution of the isolated compounds and the positive control azadirachtin at 200, 100, and 50 μL/well, with 3 replicates per treatment to the artificial diet for the newly hatched larvae, and the bioassay diet was placed into six-well plates. Newly hatched larvae were incubated at 25 °C and a relative humidity of 80%. DMSO was used as the negative control, azadirachtin was used as the positive control, and an artificial diet was used as the blank control. The number of dead larvae was recorded on the second, fourth, sixth, and eighth day after treatment.

## 4. Conclusions

Five new compounds, including two cyclopiane diterpenes conidiogenones J and K (**1**–**2**), a steroid andrastin H (**5**), an alkaloid (*Z*)-4-(5-acetoxy-*N*-hydroxy-3-methylpent-2-enamido) butanoate (**6**), and an aliphatic acid (*Z*)-5-acetoxy-3-methylpent-2-enoic acid (**7**), together with ten known compounds (**3**–**4** and **8**–**15**) were isolated from the EtOAc extracts of the fermentation broth of the *L. littorea*-derived fungus *P. oxalicum* HLLG-13. Compounds **5** and **9**–**14** exhibited strong antibacterial activities against *S. epidermidis* and *C. albicans*, with MIC values ranging from 6.25 to 25 μg/ mL. Compounds **1**–**6** and **9**–**14** exhibited significant growth inhibition activities against newly hatched *H. armigera* Hubner larvae, with IC_50_ values ranging from 50 to 100 μg/ mL.

## Figures and Tables

**Table 1:** ^1^H NMR spectroscopic data (400/600 MHz) (*δ* in ppm, *J* in Hz) for **1**–**2**, **5**–**8**, and **15**.

Position	1	2	5	6	7	8	15
1			2.07, t (13.3)	1.98, s			
			1.36, t (13.3)				
2	5.94, dd (10.0, 1.2)	5.96, dd (10.0, 0.8)	1.36, m		5.77, s		6.75, d (3.2)
			1.02, m				
3	7.08, dd (10.0, 5.6)	7.13, dd (10.0, 6.0)	3.17, m	4.11, t (7.2)		3.42, d (8.5)	
4	2.79, m	2.74, m		2.79, m	2.95, t (6.8)		
5			1.76, m		4.21, t (6.8)		6.80, d (8.8)
6	2.41, m	2.30, dd (9.4, 5.2)	2.00, m	6.30, br s		6.48, d (8.2)	6.70, dd (3.2, 8.8)
			1.53, d (13.2)				
7	1.63, m	1.57, m	2.93, t (13.2)		2.01, s	6.55, dd (2.0, 8.2)	3.77, s
	1.23, m	1.21, m	2.06, t (13.2)				
8	2.06, m	2.04, m		3.53, t (6.8)	1.95, br s		5.06, s
	1.73, m	1.72, m					
9			2.00, q (2.7)	1.77, m		6.41, d (2.0)	
10	1.74, m	2.06, m		2.30, t (7.6)		3.06, d (7.2)	2.07, s
		1.68, d (14.4)					
11			5.27, s			5.06, m	
12	2.18, d (14.8)	2.02, m		3.58, s			
	1.49, d (14.8)	1.66, m					
13	4.08, dd (10.4, 7.2)	3.94, dd (9.7, 8.6)		1.87, br s		1.57, s	
14						1.66, s	
15	1.64, d (6.1)	1.52, d (5.2)					
16	1.26, d (7.3)	1.24, d (7.2)				7.61, d (8.7)	
17	1.20, s	1.16, s				6.88, d (8.7)	
18	1.12, s	1.35, s	0.89, s				
19	1.12, s	0.98, s	0.67, s			6.88, d (8.7)	
20	0.98, s	1.00, s	1.11, s			7.61, d (8.7)	
21			10.07, s			3.78, s	
22			1.65, s				
23			1.02, s				
24							
25			3.49, s				
26			1.50, s				

**Table 2:** ^13^C NMR spectroscopic data (100/150MHz) for **1**–**2**, **5**–**8**, and **15**.

Position	1	2	5	6	7	8	15
1	208.0, C	208.2, C	27.0, CH_2_	20.7, CH_3_	169.4, C	171.7, C	152.2, C
2	128.0, CH	128.0, CH	25.8, CH_2_	170.2, C	119.5, CH	86.9, C	117.3, CH
3	157.1, CH	157.2, CH	73.5, CH	62.4, CH_2_	157.1, C	39.6, CH_2_	126.5, C
4	40.2, CH	45.8, CH	37.4, C	31.9, CH_2_	33.3, CH_2_	125.0, C	152.1, C
5	61.9, C	59.8, C	46.4, CH	149.5, C	63.8, CH_2_	155.0, C	113.1, CH
6	55.8, CH	49.9, CH	16.5, CH_2_	117.6, CH	172.7, C	115.0, CH	116.3, CH
7	35.6, CH_2_	35.5, CH_2_	32.2, CH_2_	166.2, C	20.8, CH_3_	129.8, CH	56.6, CH_3_
8	40.0, CH_2_	40.0, CH_2_	41.2, C	46.2, CH_2_	25.7, CH_3_	128.4, C	62.7, CH_2_
9	58.7, C	58.7, C	53.1, CH	21.8, CH_2_		132.4, CH	172.8, C
10	50.1, CH_2_	47.8, CH_2_	51.7, C	30.4, CH_2_		28.7, CH_2_	20.8, CH_3_
11	36.6, C	38.9, C	122.0, CH	173.0, C		123.5, CH	
12	43.5, CH_2_	47.7, CH_2_	135.5, C	51.2, CH_3_		133.0, C	
13	78.5, CH	80.8, CH	55.7, C	25.2, CH_3_		17.8, CH_3_	
14	57.4, C	57.6, C	66.9, C			25.9, CH_3_	
15	74.0, CH	73.9, CH	186.2, C			128.4, C	
16	18.8, CH_3_	19.2, CH_3_	111.5, C			130.3, CH	
17	21.5, CH_3_	21.5, CH_3_	195.5, C			116.6, CH	
18	35.1, CH_3_	33.8, CH_3_	27.4, CH_3_			159.2, C	
19	23.1, CH_3_	24.1, CH_3_	21.3, CH_3_			116.6, CH	
20	29.5, CH_3_	24.8, CH_3_	19.1, CH_3_			130.3, CH	
21			206.0, CH			53.8, CH_3_	
22			19.7, CH_3_				
23			15.5, CH_3_				
24			170.8, C				
25			51.6, CH_3_				
26			6.9, CH_3_				

**Table 3:** Antibacterial activity of compounds **5** and **9**–**14**.

Compounds	MIC (μg/ mL)	
	*S. epidermidis*	*C. albicans*
**5**	12.5	6.25
**9**	6.25	6.25
**10**	25	25
**11**	12.5	25
**12**	>50	25
**13**	12.5	6.25
**14**	6.25	6.25
Ciprofloxacin ^a^	0.313	0.313

^a^ Ciprofloxacin was used as a positive control.

**Table 4:** Growth inhibition activities of **1**–**6** and **9**–**14** against newly hatched *H. armigera* Hubner larvae.

Compounds	IC_50_ (μg/ mL)
**1**	200
**2**	200
**3**	100
**4**	100
**5**	50
**6**	200
**9**	200
**10**	100
**11**	200
**12**	100
**13**	200
**14**	200
Azadirachtin ^b^	50

^b^ Azadirachtin was used as a positive control.

## Supplementary Materials

The following are available online: https://www.mdpi.com/article/10.3390/md21010022/s1, Figures S1–S54: HR-ESI-MS, 1D and 2D NMR spectra of compounds **1**, **2**, **5**, **6**, **7**, **8** and **15**. S55: 1H and 13C NMR data of the known compounds **3**–**4**, **9**–**14**.

## References

[1] Darwish A.G.G., Samy M.N., Sugimoto S., Otsuka H., Abdel-Salam H., Matsunami K. (2016). Effects of Hepatoprotective Compounds from the Leaves of *Lumnitzera racemosa* on Acetaminophen-Induced Liver Damage in Vitro. Chem. Pharm. Bull..

[2] Ahmed G.G.D., Mamdouh N.S., Sachiko S., Hideaki O., Hosni A.S., Katsuyoshi M. (2019). A New macrolactone, racemolide along with seven known compounds with biological activities from mangrove plant, *Lumnitzera racemose*. Nat. Prod. Commun..

[3] Nguyen P.T., Bui T.T.L., Chau N.D., Bui H.T., Eun J.K., Hee K.K., Sang H.L., Hae D.J., Nguyen T.C., Nguyen V.T. (2015). In vitro evaluation of the antioxidant and cytotoxic activities of constituents of the mangrove *Lumnitzera racemosa* Willd. Arch. Pharm. Res..

[4] Yu S.Y., Wang S.W., Hwang T.L., Wei B.L., Su C.J., Chang F.R., Cheng Y.B. (2018). Components from the Leaves and Twigs of Mangrove *Lumnitzera racemosa* with Anti-Angiogenic and Anti-Inflammatory Effects. Mar. Drugs.

[5] Meng L.H., Li X.M., Liu Y., Wang B.G. (2014). Penicibilaenes A and B, sesquiterpenes with a tricyclo[6.3.1.0^1,5^]dodecane skeleton from the marine isolate of *Penicillium bilaiae* MA-267. Org. Lett..

[6] Meng L.H., Li X.M., Lv C.T., Li C.S., Xu G.M., Huang C.G., Wang B.G. (2013). Sulfur-containing cytotoxic curvularin macrolides from *Penicillium sumatrense* MA-92, a fungus obtained from the rhizosphere of the mangrove *Lumnitzera racemose*. J. Nat. Prod..

[7] Qin Y.Y., Zou L.H., Lei X.G., Su J.W., Yang R.X., Xie W.J., Li W.S., Chen G.Y. (2023). OSMAC strategy integrated with molecular networking discovery peniciacetals A−I, nine new meroterpenoids from the mangrove-derived fungus *Penicillium* sp. HLLG-122. Bioorg. Chem..

[8] Yang J.Y., Tang M.M., Chen L., Lai X.Y., Zhuo X., Zhou X.M., Chen G.Y. (2022). Study on the secondary metabolites of endophytic *Penicillium sclerotiorum* HLL113. Chin. J. Org. Chem..

[9] Zheng C.J., Bai M., Zhou X.M., Huang G.L., Shao T.M., Luo Y.P., Niu Z.G., Niu Y.Y., Chen G.Y., Han C.R. (2018). Penicilindoles A-C, Cytotoxic Indole Diterpenes from the Mangrove-Derived Fungus *Eupenicillium* sp. HJ002. J. Nat. Prod..

[10] Cai J., Zhu X.C., Zeng W.N., Wang B., Luo Y.P., Liu J., Chen M.J., Li G.Y., Huang G.L., Chen G.Y. (2022). Talaromarins A–F: Six New Isocoumarins from Mangrove-Derived Fungus *Talaromyces flavus* TGGP35. Mar. Drugs.

[11] Bai M., Zheng C.J., Tang D.Q., Zhang F., Wang H.Y., Chen G.Y. (2019). Two new secondary metabolites from a mangrove-derived fungus *Cladosporium* sp. JS1-2. J. Antibiot..

[12] Bai M., Huang G.L., Mei R.Q., Wang B., Luo Y.P., Nong X.H., Chen G.Y., Zheng C.J. (2019). Bioactive Lactones from the Mangrove-Derived Fungus *Penicillium* sp. TGM112. Mar. Drugs.

[13] Du L., Li D., Zhu T., Cai S., Wang F., Xiao X., Gu Q. (2009). New alkaloids and diterpenes from a deep ocean sediment derived fungus *Penicillium* sp.. Tetrahedron.

[14] Yudai M., Takayoshi A., Ikuro A. (2013). Reconstituted biosynthesis of fungal meroterpenoid andrastin A. Tetrahedron.

[15] Saima K., Muhammad I.T., Naheed R., Mamona N., Mahreen M., Liaquat A., Rasool B.T., Muhammad S.J. (2019). Rarely occurring natural products isolated from *Vincetoxicum stocksii*. Chem. Soc. Pak..

[16] Tian T., Li L.Q., Xue J.J., Zhang J., Li Y.J. (2015). Enantioselective Syntheses of Spiroketals via a Tandem Reaction of Cu(I)-Catalyzed Cycloetherification and Hydrogen-Bond-Induced [4 + 2] Cyclization. Org. Chem..

[17] Li X., Xue J.J., Huang C.S., Li Y. (2012). Copper(I)-Catalyzed hydroalkoxylation/hydrogen-bonding-induced asymmetric hetero-diels–alder cycloaddition cascade: An approach to aromatic spiroketals. Chem. Asian J..

[18] Selvanathan A., Vladimir V.P. (2010). Dual Reactivity of Hydroxy- and Methoxy- Substituted o-Quinone Methides in Aqueous Solutions: Hydration versus Tautomerization. J. Org. Chem..

[19] Bray C.D. (2008). An Approach to Benzannelated [5,6]-Spiroketals. Synlett.

[20] Wang C.F., Huang X.F., Xiao H.X., Hao Y.J., Xu L., Yan Q.X., Zou Z.B., Xie C.L., Xu Y.Q., Yang X.W. (2021). Chemical Constituents of the Marine Fungus *Penicillium* sp. MCCC 3A00228. Chem. Biodivers..

[21] Abdelhameed R.F.A., Habib E.S., Goda M.S., Fahim J.R., Hassanean H.A., Eltamany E.E., Ibrahim A.K., AboulMagd A.M., Fayez S., Abd E. (2020). Thalassosterol, a New Cytotoxic Aromatase Inhibitor Ergosterol Derivative from the Red Sea Seagrass *Thalassodendron ciliatum*. Mar. Drugs.

[22] Tettamanzi M.C., Jares E.A., Iannone L.M., Pomilio A.B. (1994). Constituents of Senecio crassiflorus. Fitoterapia.

[23] Lorensi G.H., Oliveira R.S., Leal A.P., Zanatta A.P., de Almea C.G.M., Barreto Y.C., Rosa M.E., Vieira P.B., Ramos C.J.B., Victoria F.C. (2019). Entomotoxic Activity of *Prasiola crispa* (Antarctic Algae) in *Nauphoeta cinerea* Cockroaches: Identification of Main Steroidal Compounds. Mar. Drugs.

[24] Liu S., Su M., Song S.J., Hong J., Chung H.Y., Jung J.H. (2018). An anti-inflammatory PPAR-γ agonist from the jellyfish-derived fungus *Penicillium chrysogenum* J08NF-4. J. Nat. Prod..

[25] Kusakabe K., Honmura Y., Uesugi S., Tonouchi A., Maeda H., Kimura K., Koshino H., Hashimoto M. (2017). Neomacrophorin X, a [4.4.3]Propellane-Type Meroterpenoid from *Trichoderma* sp. 1212-03. J. Nat. Prod..

[26] Zhang S.T., He Y., Li F.L., Lin S., Yang B.Y., Mo S.Y., Li H.Q., Wang J.P., Qi C.X., Hu Z.X. (2020). Bioassay-directed isolation of antibacterial metabolites from an arthropod-derived *Penicillium chrysogenum*. J. Nat. Prod..

[27] Li F.L., Sun W.G., Zhang S.T., Gao W.X., Lin S., Yang B.Y., Chai C.W., Li H.Q., Wang J.P., Hu Z.X. (2020). New cyclopiane diterpenes with anti-inflammatory activity from the sea sediment-derived fungus *Penicillium* sp. TJ403-2. Chin. Chem. Lett..

[28] Mazlan N.W., Clements C., Edrada E.R.A. (2020). Targeted Isolation of Anti-Trypanosomal Naphthofuran-Quinone Compounds from the Mangrove Plant *Avicennia lanata*. Mar. Drugs.

[29] Cheng Z.B., Li Y.L., Xu W., Liu W., Liu L.J., Zhu D.G., Kang Y., Luo Z.H., Li Q. (2019). Three new cyclopiane-type diterpenes from a deep-sea derived fungus *Penicillium* sp. YPGA11 and their effects against human esophageal carcinoma cells. Bioorg. Chem..

[30] Xie C.L., Zhang D., Xia J.M., Hu C.C., Lin T., Lin Y.K., Wang G.H., Tian W.J., Li Z.P., Zhang X.K. (2019). Steroids from the Deep-Sea-Derived Fungus *Penicillium granulatum* MCCC 3A00475 Induced Apoptosis via Retinoid X Receptor (RXR)-α Pathway. Mar. Drugs.

[31] Pierce C.G., Uppuluri P., Tristan A.R., Wormley F.L., Mowat E., Ramage G., Lopez-Ribot J.L. (2008). A simple and reproducible 96-well plate-based method for the formation of fungal biofilms and its application to antifungal susceptibility testing. Nat. Protoc..

[32] Guo Z.K., Gai C.J., Cai C.H., Chen L.L., Liu S.B., Zeng Y.B., Yuan J.Z., Mei W.L., Dai H.F. (2017). Metabolites with Insecticidal Activity from *Aspergillus fumigatus* JRJ111048 Isolated from Mangrove Plant *Acrostichum specioum* Endemic to Hainan Island. Mar. Drugs.

