# Supplementary material for: Biological Secondary Metabolites from the Lumnitzera littorea-Derived Fungus Penicillium oxalicum HLLG-13
Source: Mar Drugs. 2022 Dec 27;21(1):22. doi: 10.3390/md21010022 (PMC9860879; doi:10.3390/md21010022)
Supplement: Supplementary file 1 [file marinedrugs-21-00022-s001.zip › marinedrugs-2123942-supplementary.pdf]

SUPPLEMENTARY MATERIALS

Biological Secondary Metabolites from the *Lumnitzera littorea*-Derived Fungus *Penicillium oxalicum* HLLG-13

Yue Wang [1,2,†], Wenhao Chen [1,2,†], Zhefei Xu [1,2], Qiqi Bai [1,2], Xueming Zhou [1,2], Caijuan Zheng [1,2], Meng Bai [3] and Guangying Chen [1,2,*]

[1] Key Laboratory of Tropical Medicinal Resource Chemistry of Ministry of Education, College of Chemistry and Chemical Engineering, Hainan Normal University, Haikou 571158, China
[2] Key Laboratory of Tropical Medicinal Plant Chemistry of Hainan Province, Haikou 571158, China
[3] Guangxi Key Laboratory of Marine Drugs, Institute of Marine Drugs, Guangxi University of Chinese Medicine, Nanning 530200, China
* Correspondence: chgying123@163.com
† These authors contributed equally to this work.

Contents

Figure S1. HR-ESI-MS spectrum of **1** 4

Figure S2. ^{1}H NMR spectrum of **1** in methanol-d_4 4

Figure S3. ^{13}C NMR spectrum of **1** in methanol-d_4 5

Figure S4. 135-DEPT spectrum of **1** in methanol-d_4 6

Figure S5. HSQC spectrum of **1** in methanol-d_4 6

Figure S6. ^{1}H-^{1}H COSY spectrum of **1** in methanol-d_4 7

Figure S7. HMBC spectrum of **1** in methanol-d_4 7

Figure S8. NOESY spectrum of **1** in methanol-d_4 8

Figure S9. HR-ESI-MS spectrum of **2** 8

Figure S10. ^{1}H NMR spectrum of **2** in methanol-d_4 9

Figure S11. ^{13}C NMR spectrum of **2** in methanol-d_4 9

Figure S12. 135-DEPT spectrum of **2** in methanol-d_4 10

Figure S13. HSQC spectrum of **2** in methanol-d_4 methanol-d_4 10

Figure S14. ^{1}H-^{1}H COSY spectrum of **2** in methanol-d_4 11

Figure S15. HMBC spectrum of **2** in methanol-d_4 11

Figure S16. NOESY spectrum of **2** in methanol-d_4 12

Figure S17. HR-ESI-MS spectrum of **5** 12

Figure S18. ^{1}H NMR spectrum of **5** in DMSO-d_6 13

Figure S19. ^{13}C NMR spectrum of **5** in DMSO-d_6 13

Figure S20. 135-DEPT spectrum of **5** in DMSO-d_6 14

Figure S21. HSQC spectrum of **5** in DMSO-d_6 14

Figure S22. ^{1}H-^{1}H COSY spectrum of **5** in DMSO-d_6 15

Figure S23. HMBC spectrum of **5** in DMSO-d_6 15

Figure S24. NOESY spectrum of **5** in DMSO-d_6 16

Figure S25. HR-ESI-MS spectrum of **6** 16

Figure S26. ^{1}H NMR spectrum of **6** in DMSO-d_6 17

Figure S27. ^{13}C NMR spectrum of **6** in DMSO-d_6 17

Figure S28. 135-DEPT spectrum of **6** in DMSO-d_6 18

Figure S29. HSQC spectrum of **6** in DMSO-d_6 18

Figure S30. ^{1}H-^{1}H COSY spectrum of **6** in DMSO-d_6 19

Figure S31. HMBC spectrum of **6** in DMSO-d_6 19

Figure S32. NOESY spectrum of **6** in DMSO-d_6 20

Figure S33. HR-ESI-MS spectrum of **7** 20

Figure S34. ^{1}H NMR spectrum of **7** in DMSO-d_6 21

Figure S35. ^{13}C NMR spectrum of **7** in DMSO-d_6 21

Figure S36. 135-DEPT spectrum of **7** in DMSO-d_6 22

Figure S37. HSQC spectrum of **7** in DMSO-d_6 22

Figure S38. ^{1}H-^{1}H COSY spectrum of **7** in DMSO-d_6 23

Figure S39. HMBC spectrum of **7** in DMSO-d_6 23

Figure S40. NOESY spectrum of **7** in DMSO-d_6 24

Figure S41. HR-ESI-MS spectrum of **8** 24

Figure S42. ^{1}H NMR spectrum of **8** in methanol-d_4 25

Figure S43. ^{13}C NMR spectrum of **8** in methanol-d_4 25

Figure S44. 135-DEPT spectrum of **8** in methanol-d_4 26

Figure S45. HSQC spectrum of **8** in methanol-d_4 26

Figure S46. ^{1}H-^{1}H COSY spectrum of **8** in methanol-d_4 27

Figure S47. HMBC spectrum of **8** in methanol-d_4 27

Figure S48. HR-ESI-MS spectrum of **15** 28

Figure S49. ^{1}H NMR spectrum of **15** in methanol-d_4 28

Figure S50. ^{13}C NMR spectrum of **15** in methanol-d_4 29

Figure S51. 135-DEPT spectrum of **15** in methanol-d_4 29

Figure S52. HSQC spectrum of **15** in methanol-d_4 30

Figure S53. ^{1}H-^{1}H COSY spectrum of **15** in methanol-d_4 30

Figure S54. HMBC spectrum of **15** in methanol-d_4 31

S55. ^{1}H and ^{13}C NMR data of compounds **3-4**, **9-14** 32

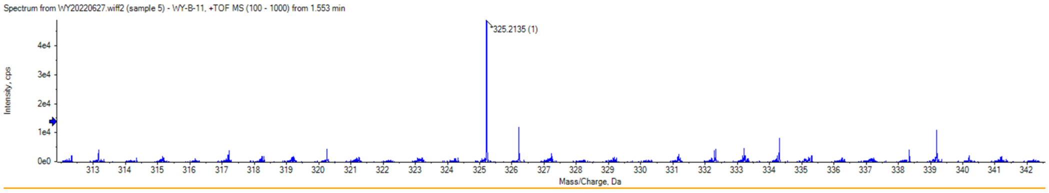

Figure S1. HR-ESI-MS spectrum of **1**

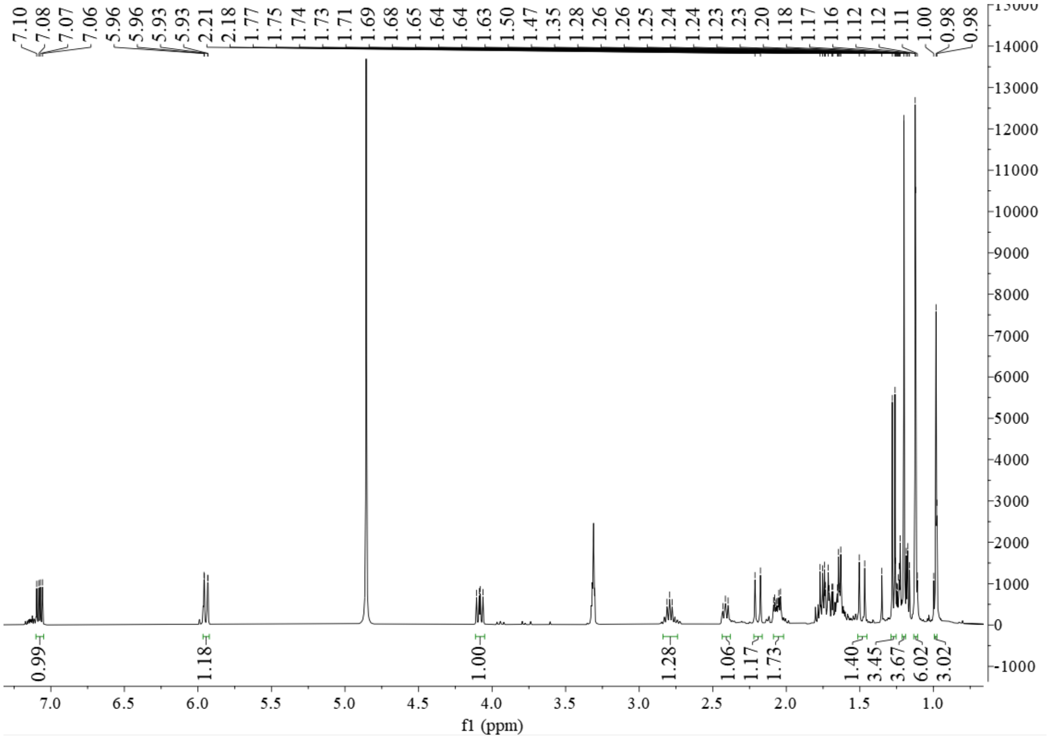

Figure S2. ^{1}H NMR spectrum of **1** in methanol-d_4

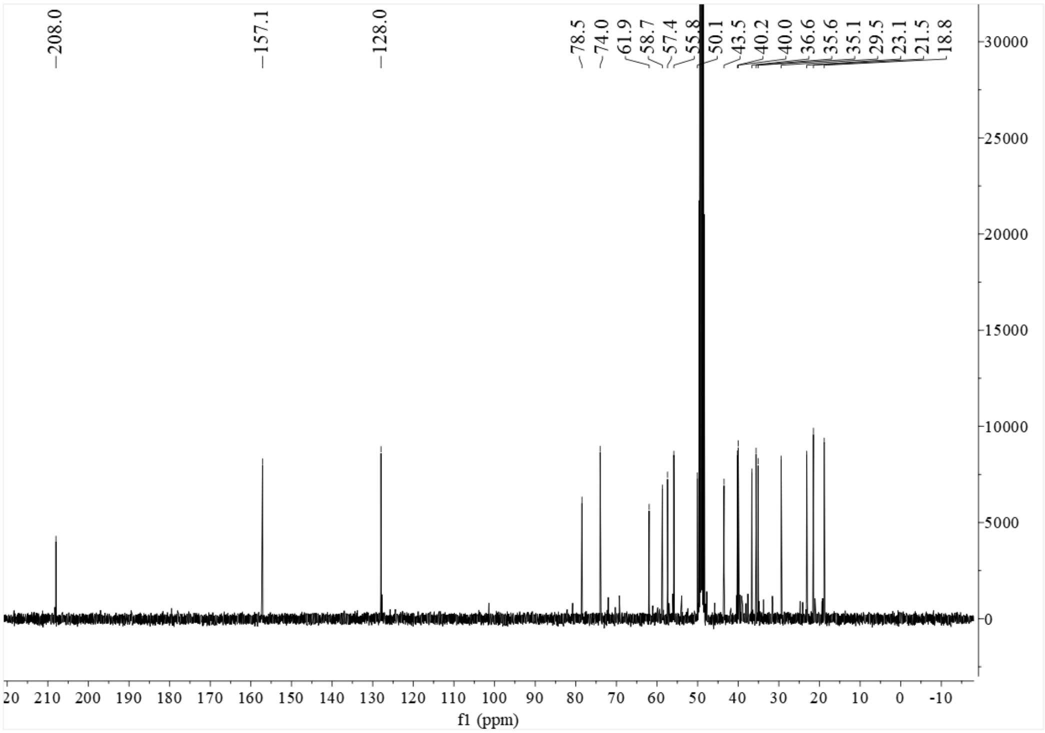

Figure S3. ^{13}C NMR spectrum of **1** in methanol-d_4

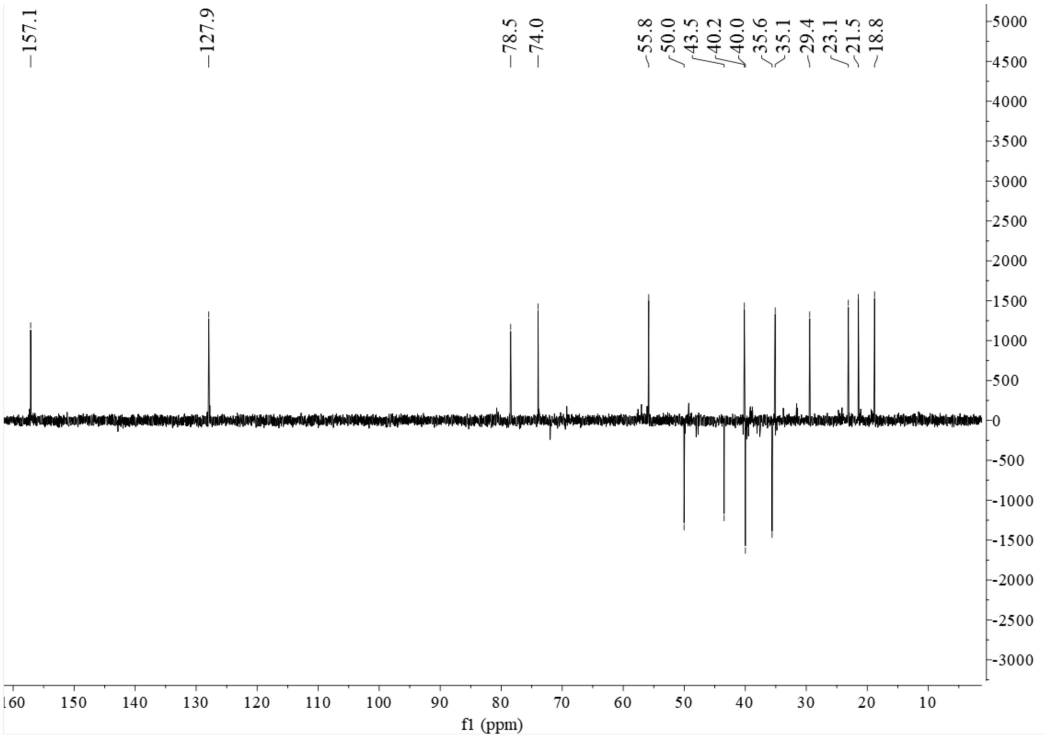

Figure S4. 135-DEPT spectrum of **1** in methanol-d_4

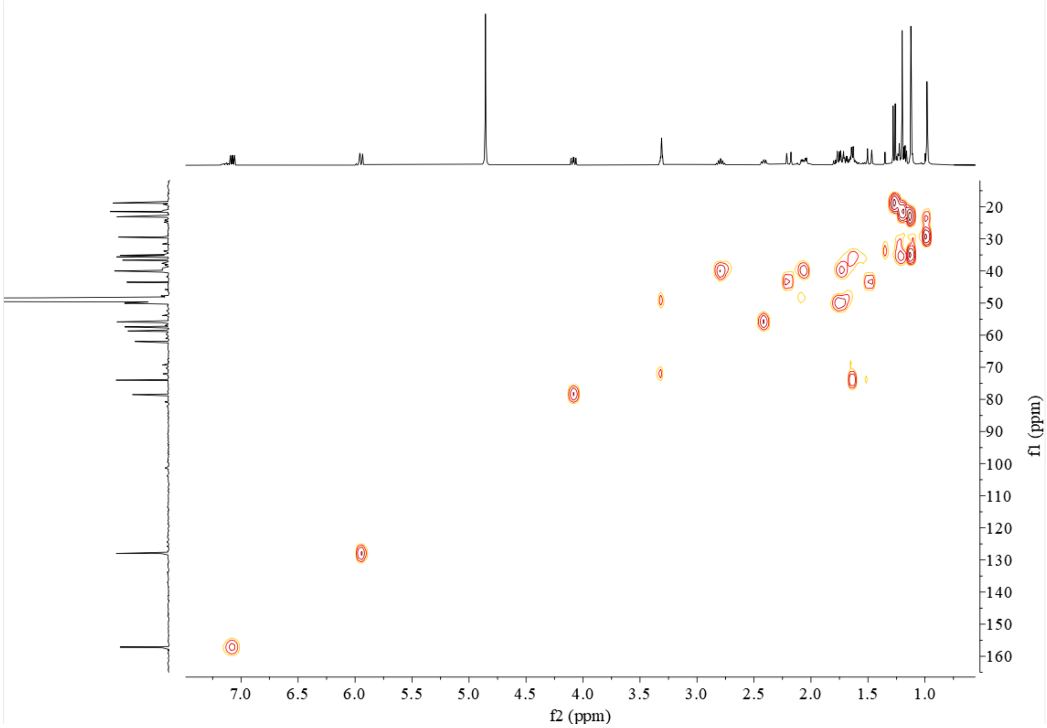

Figure S5. HSQC spectrum of **1** in methanol-d_4

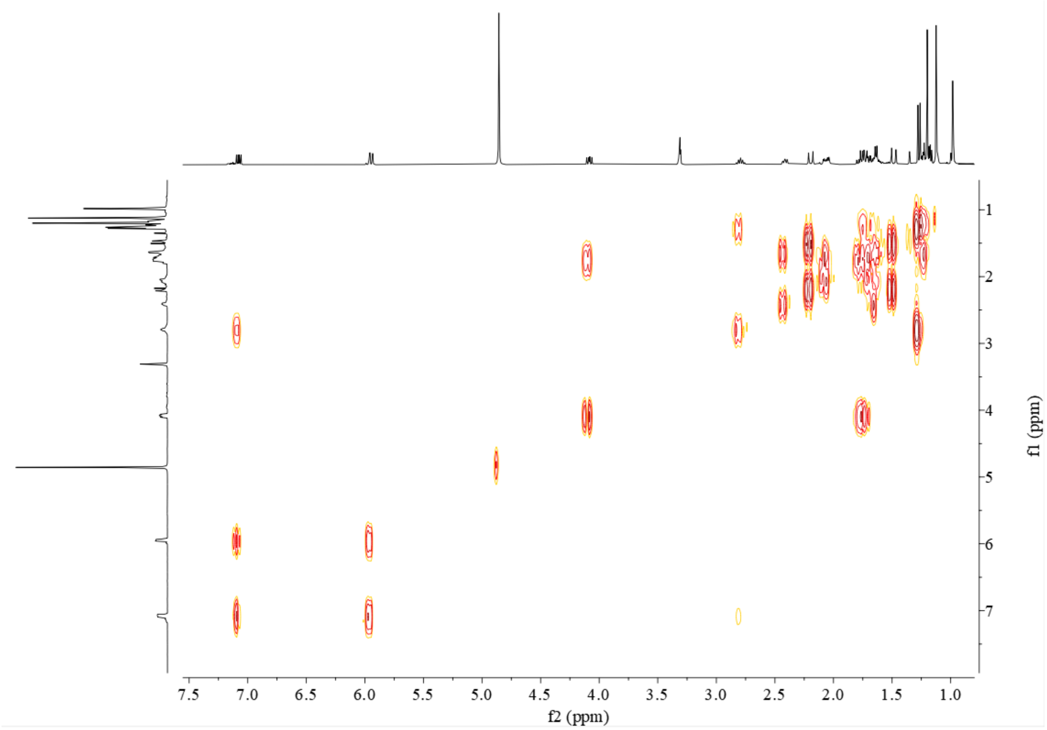

Figure S6. ^{1}H-^{1}H COSY spectrum of **1** in methanol-d_4

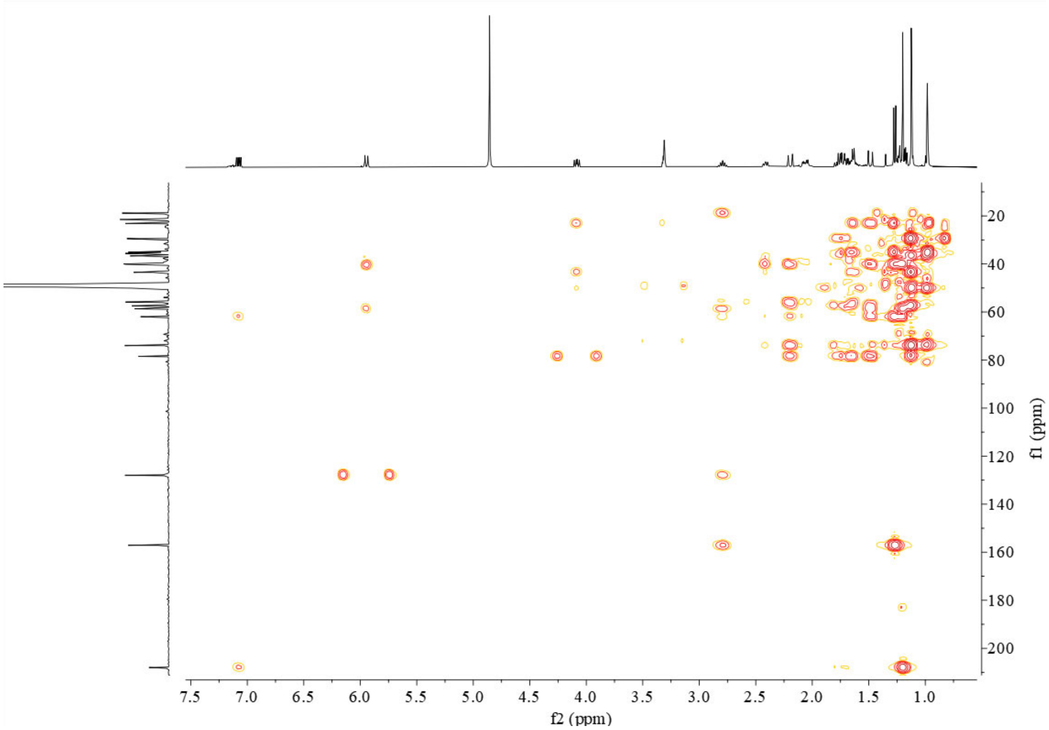

Figure S7. HMBC spectrum of **1** in methanol-d_4

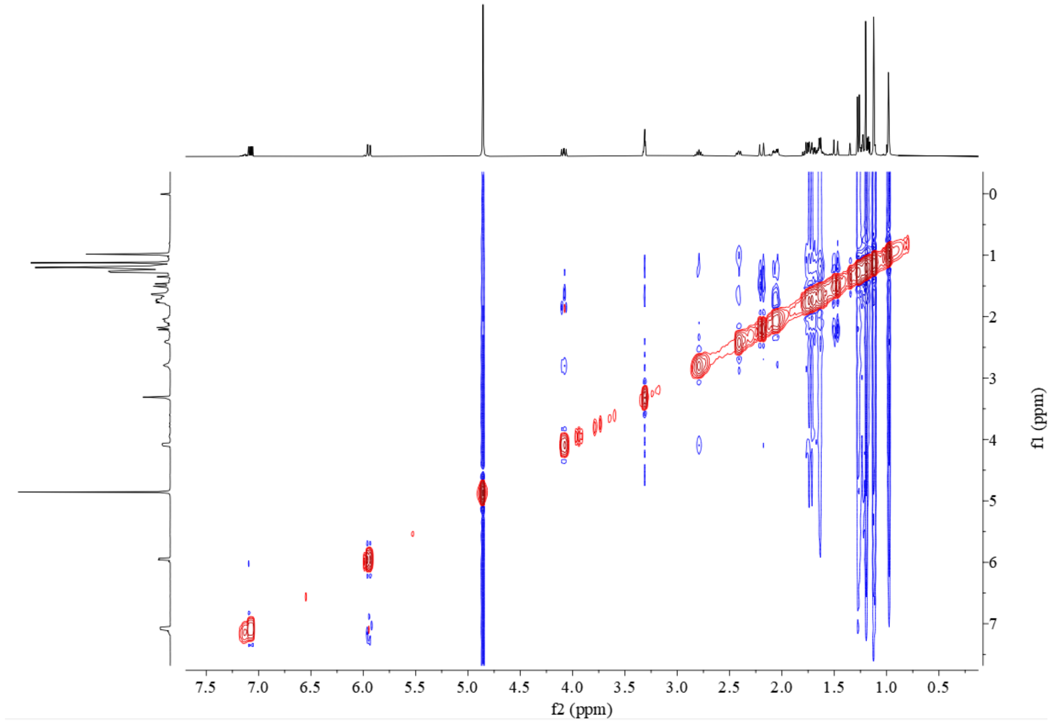

Figure S8. NOESY spectrum of **1** in methanol-d_4

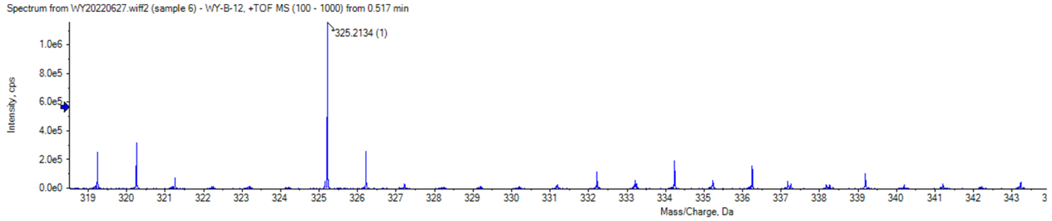

Figure S9. HR-ESI-MS spectrum of **2**

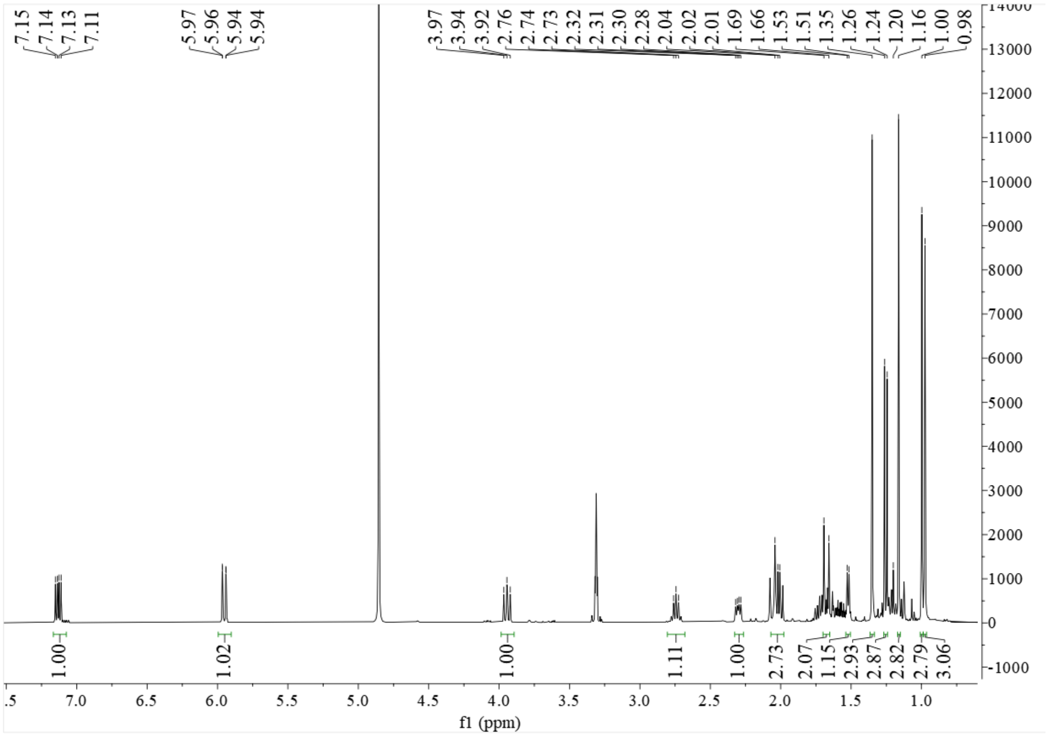

Figure S10. ^{1}H NMR spectrum of **2** in methanol-d_4

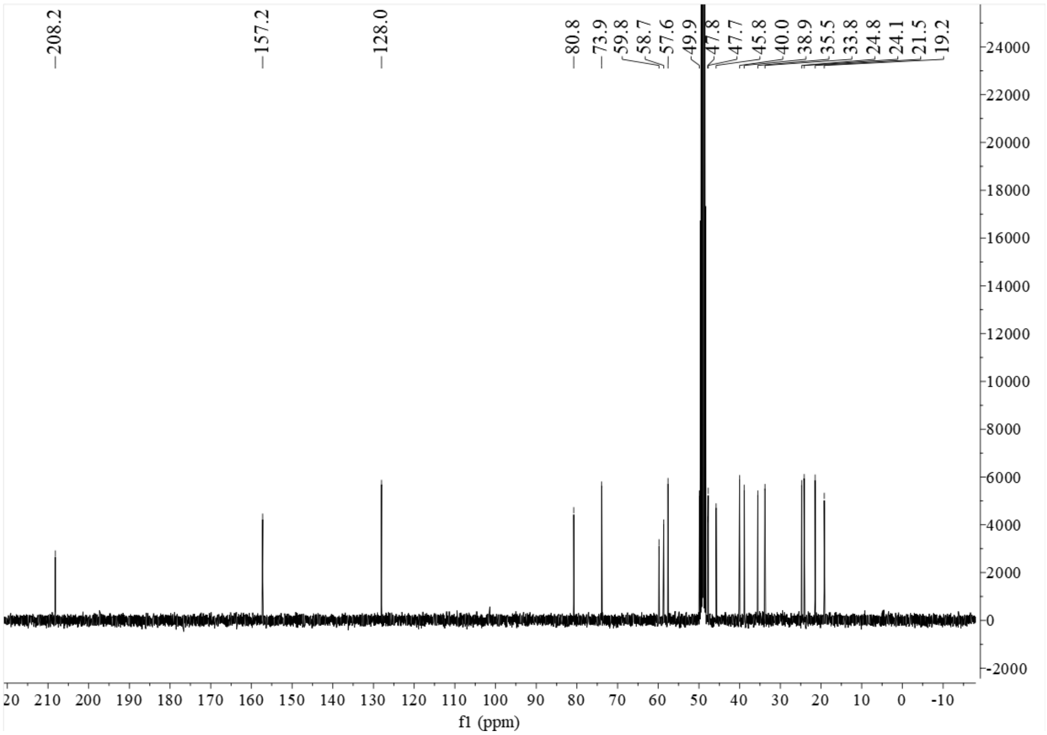

Figure S11. ^{13}C NMR spectrum of **2** in methanol-d_4

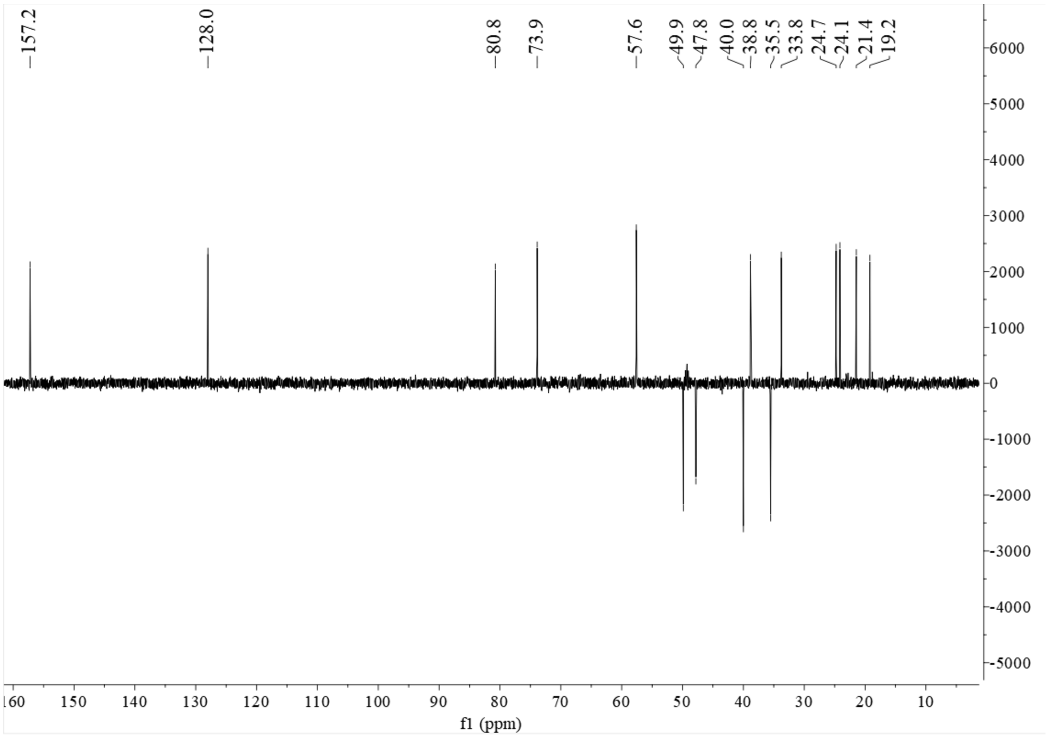

Figure S12. 135-DEPT spectrum of **2** in methanol-d_4

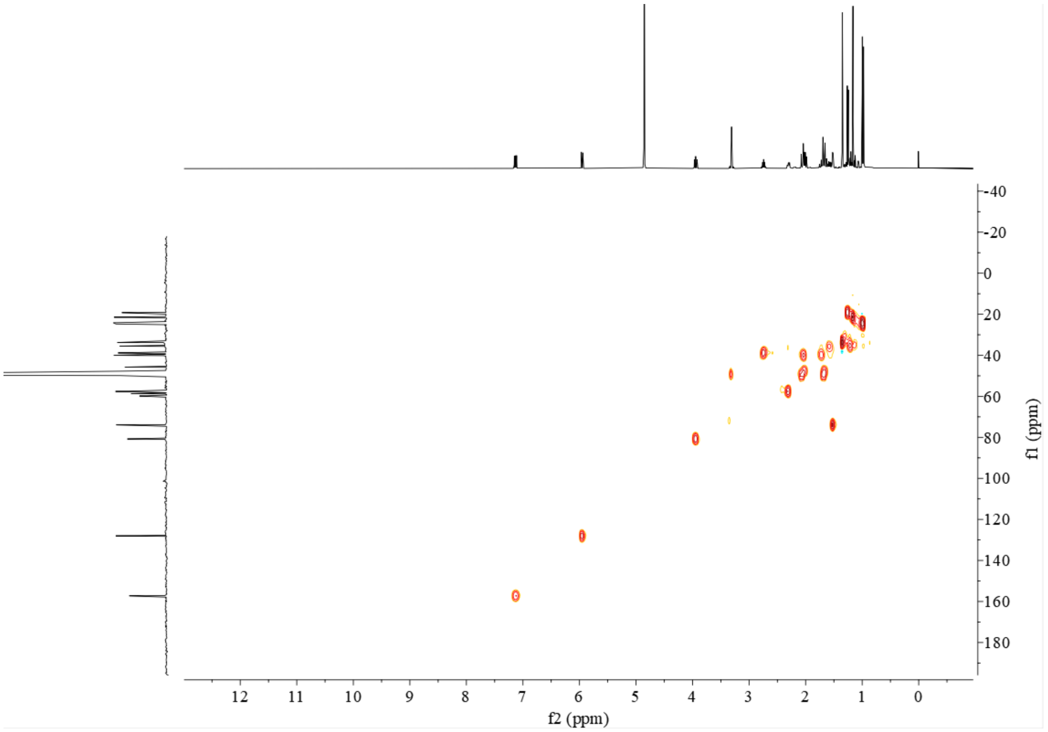

Figure S13. HSQC spectrum of **2** in methanol-d_4 methanol-d_4

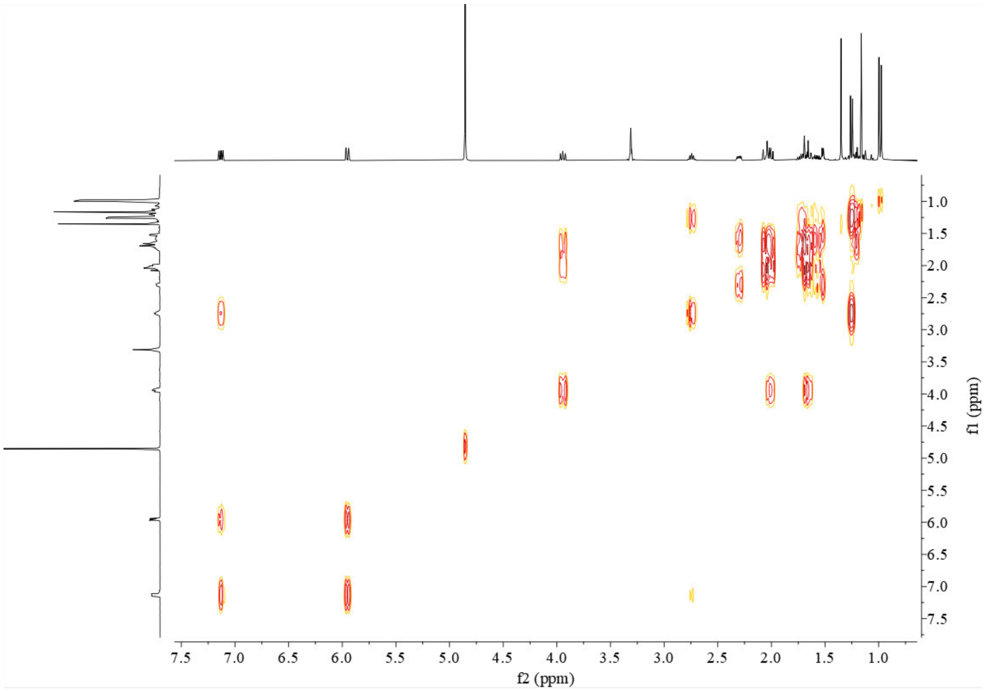

Figure S14. ^{1}H-^{1}H COSY spectrum of **2** in methanol-d_4

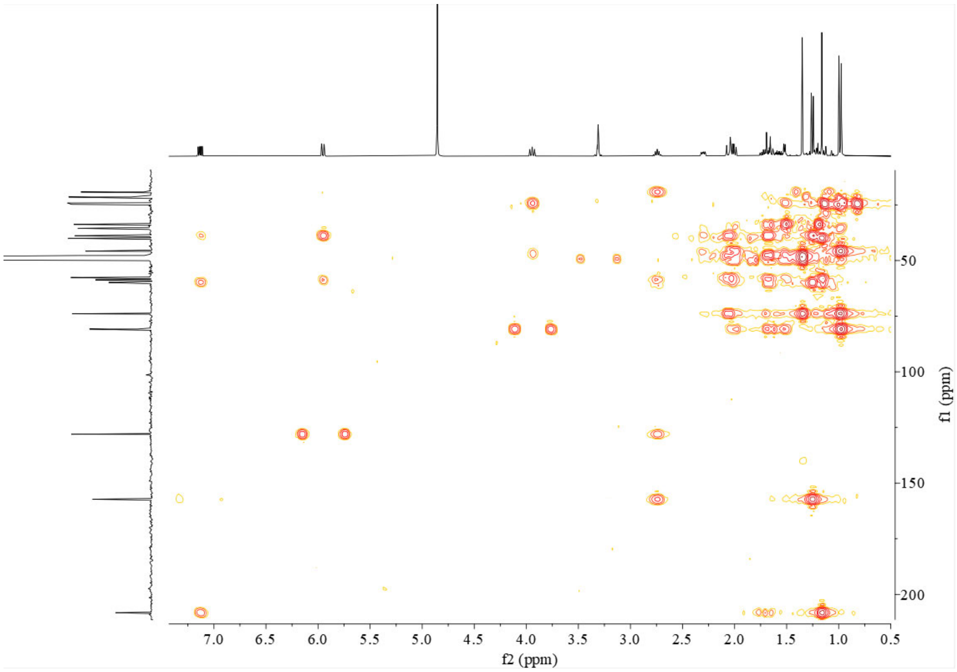

Figure S15. HMBC spectrum of **2** in methanol-d_4

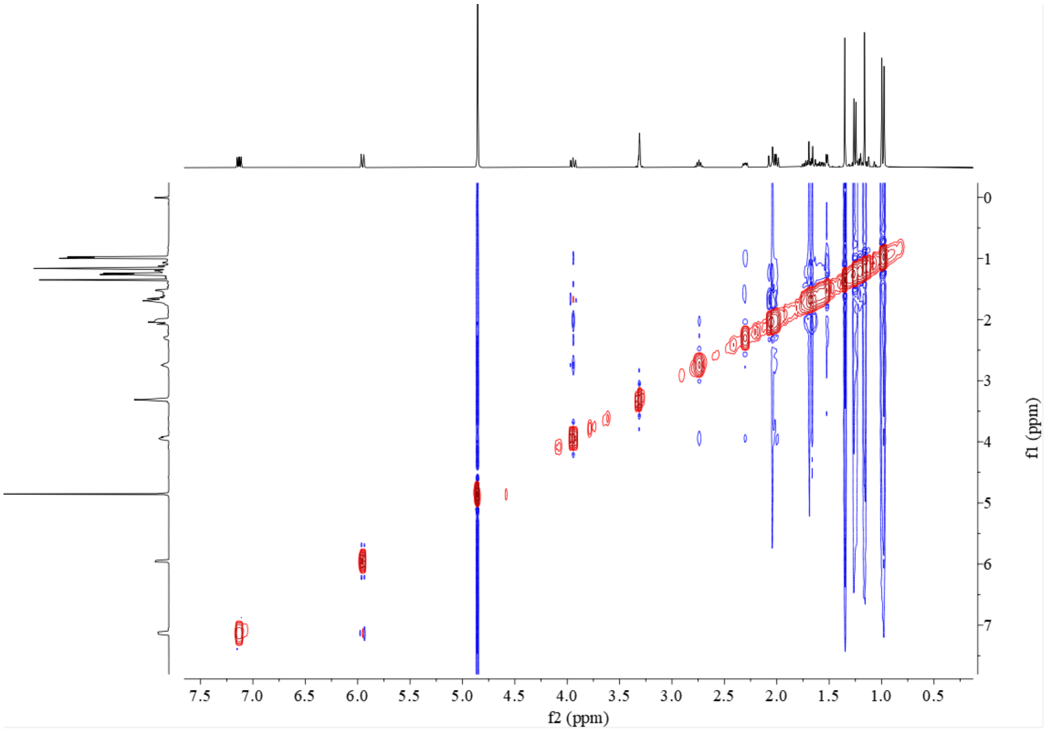

Figure S16. NOESY spectrum of **2** in methanol-d_4

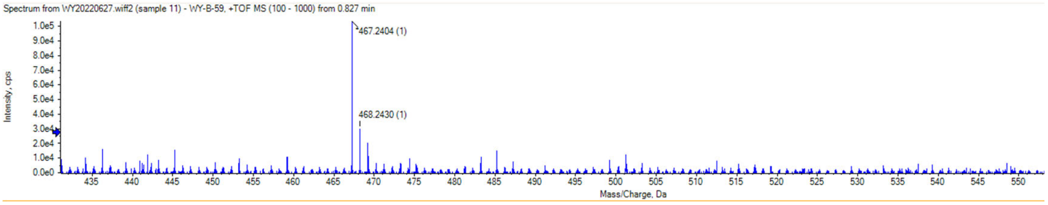

Figure S17. HR-ESI-MS spectrum of **5**

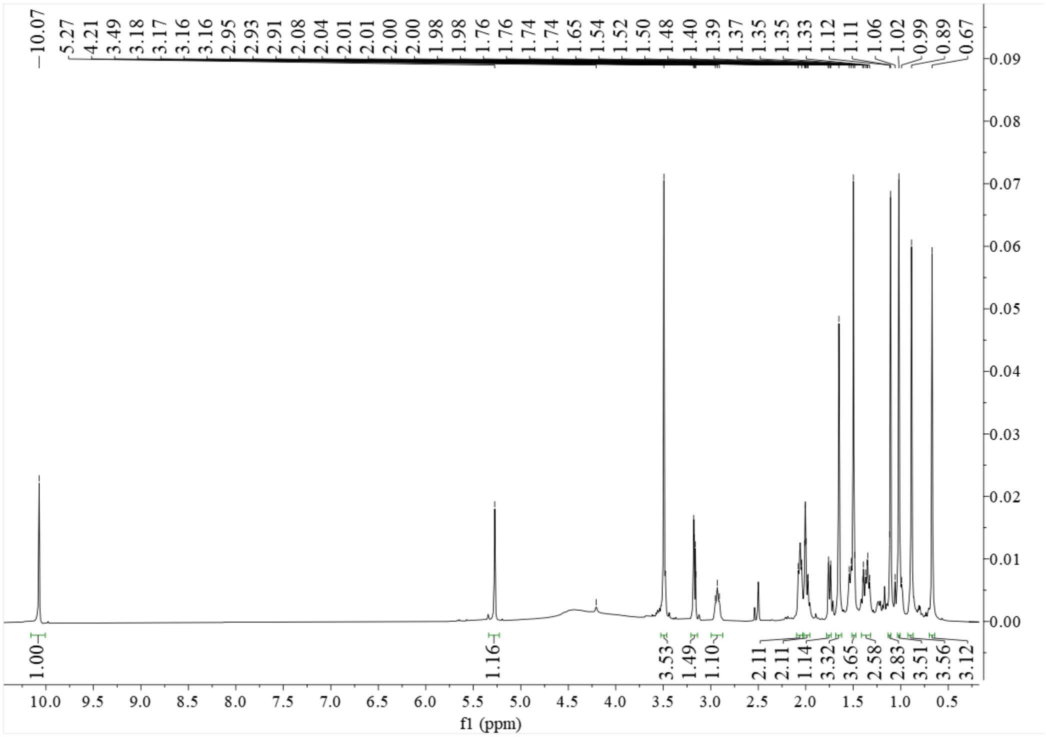

Figure S18. ^{1}H NMR spectrum of **5** in DMSO-d_6

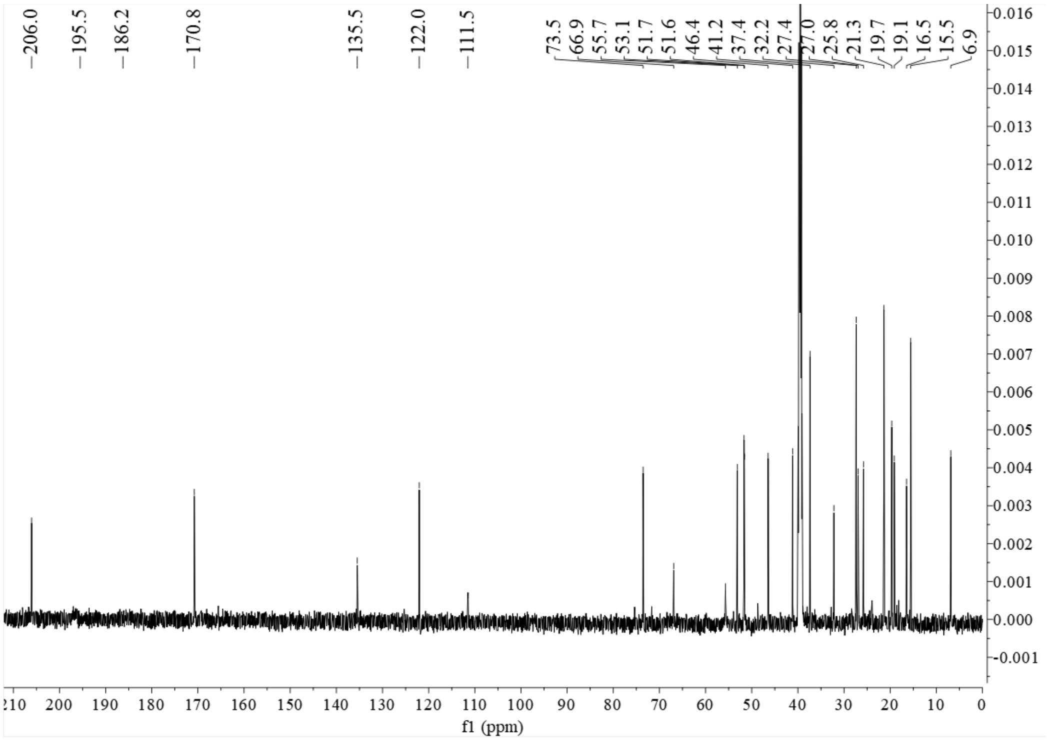

Figure S19. ^{13}C NMR spectrum of **5** in DMSO-d_6

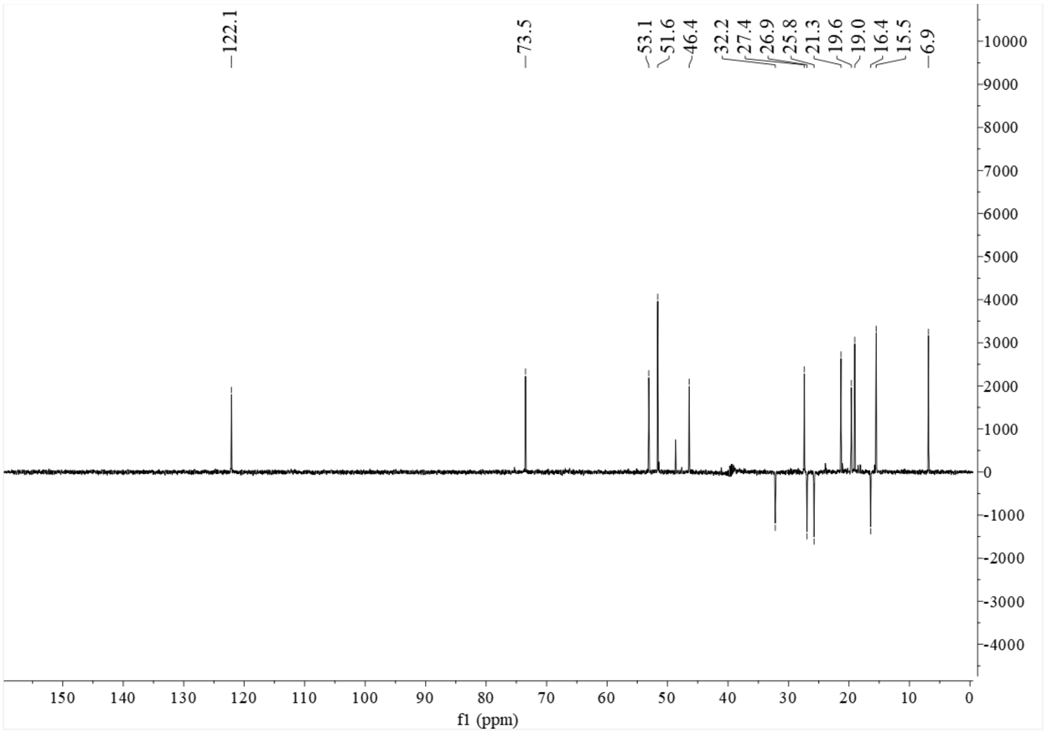

Figure S20. 135-DEPT spectrum of **5** in DMSO-d_6

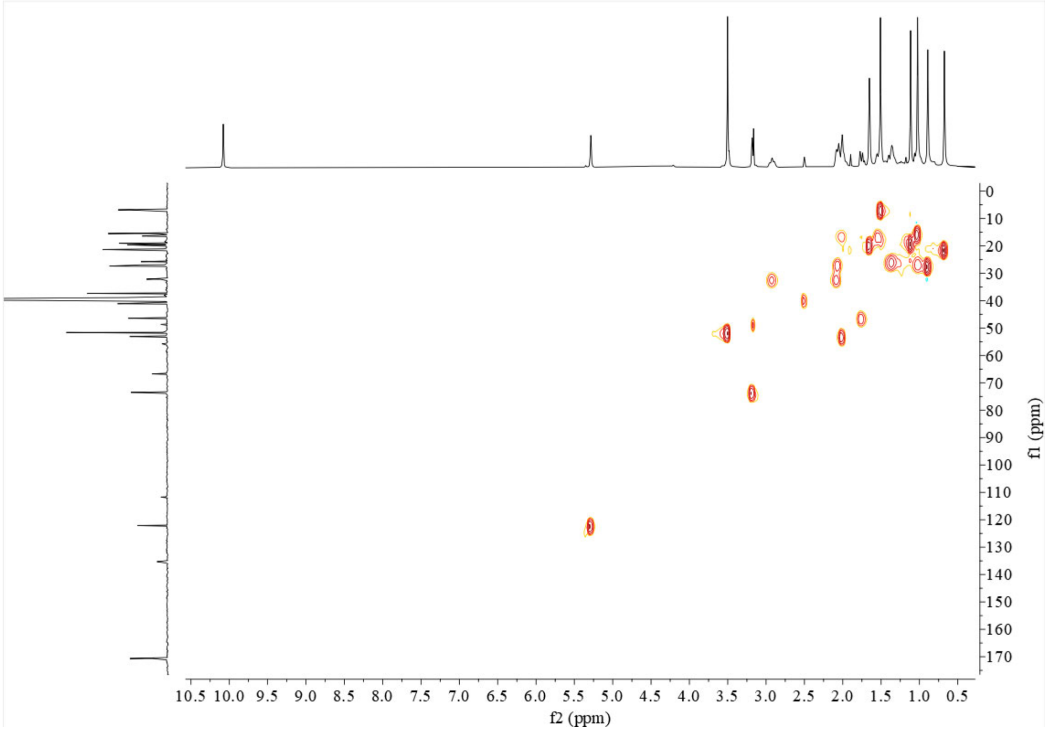

Figure S21. HSQC spectrum of **5** in DMSO-d_6

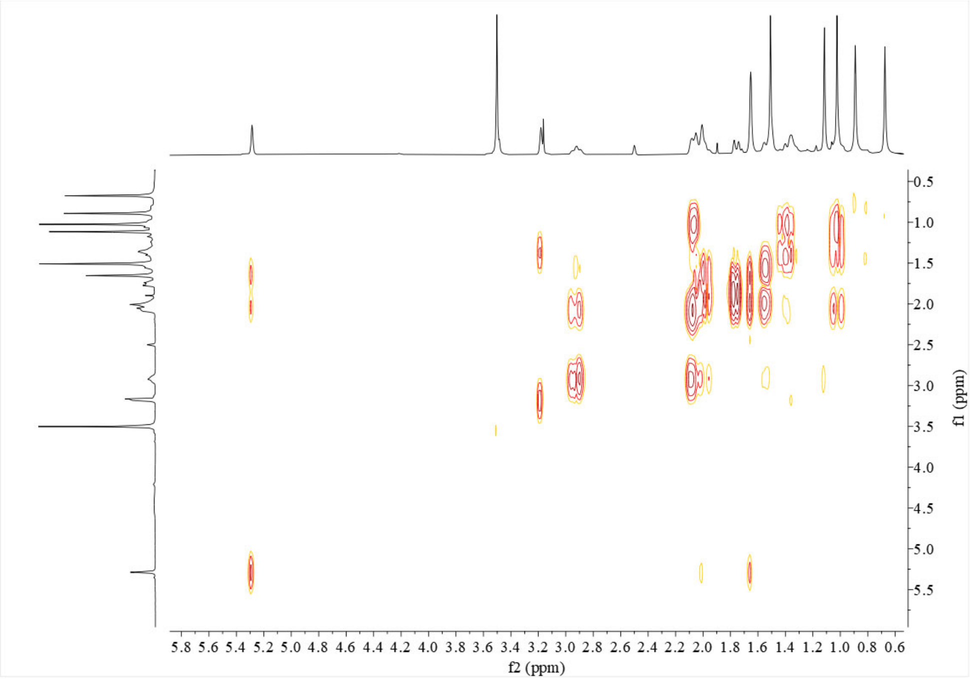

Figure S22. ^{1}H-^{1}H COSY spectrum of **5** in DMSO-d_6

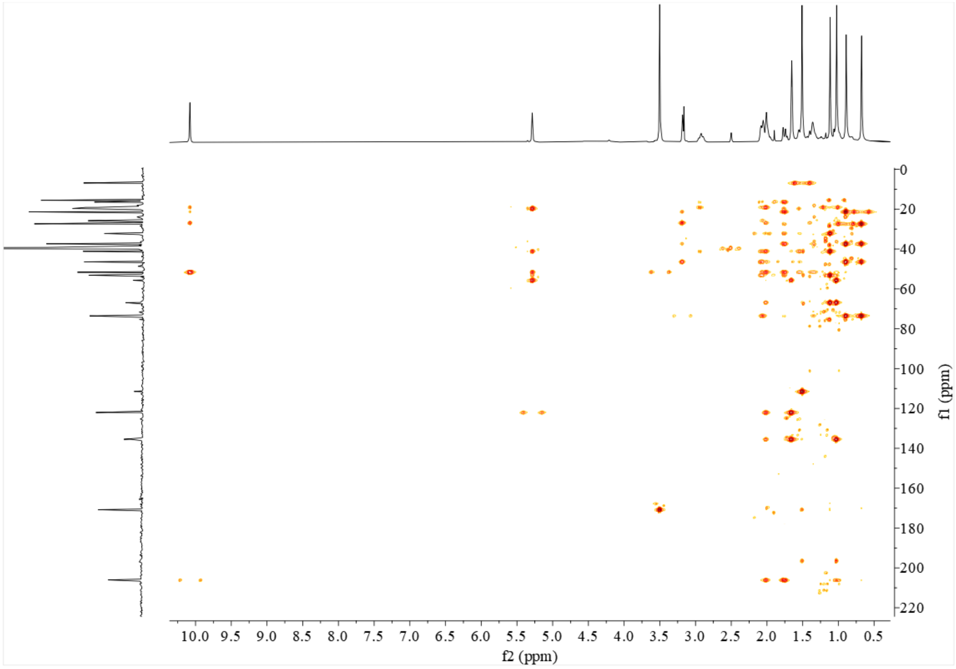

Figure S23. HMBC spectrum of **5** in DMSO-d_6

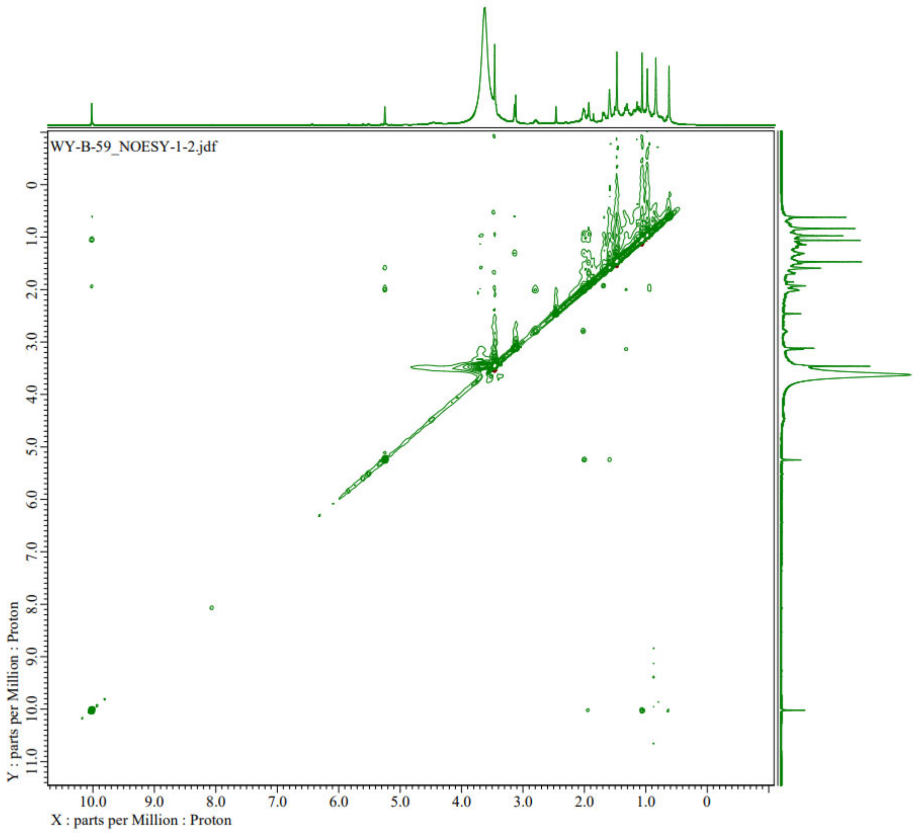

Figure S24. NOESY spectrum of **5** in DMSO-d_6

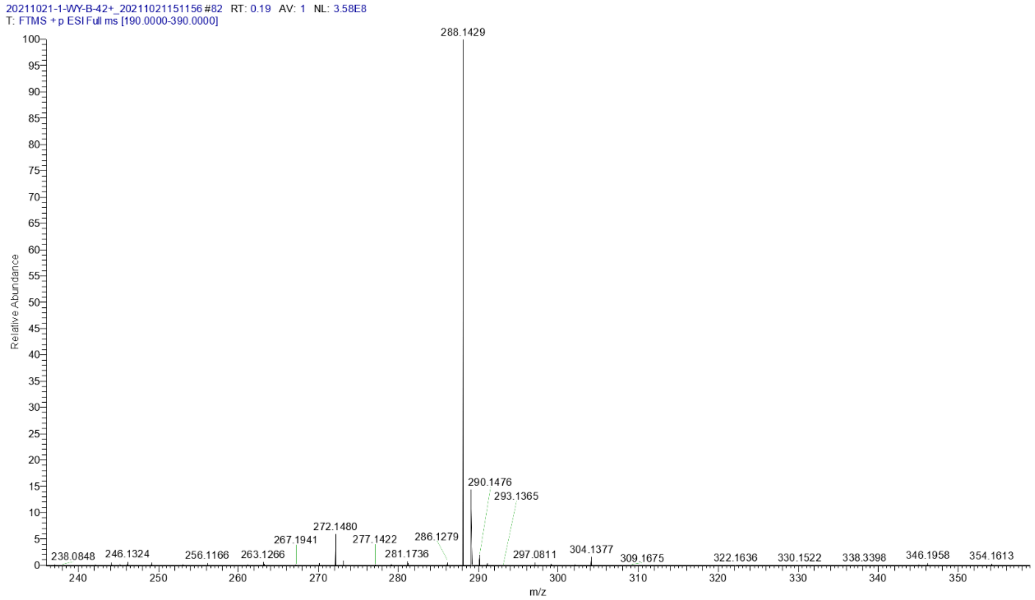

Figure S25. HR-ESI-MS spectrum of **6**

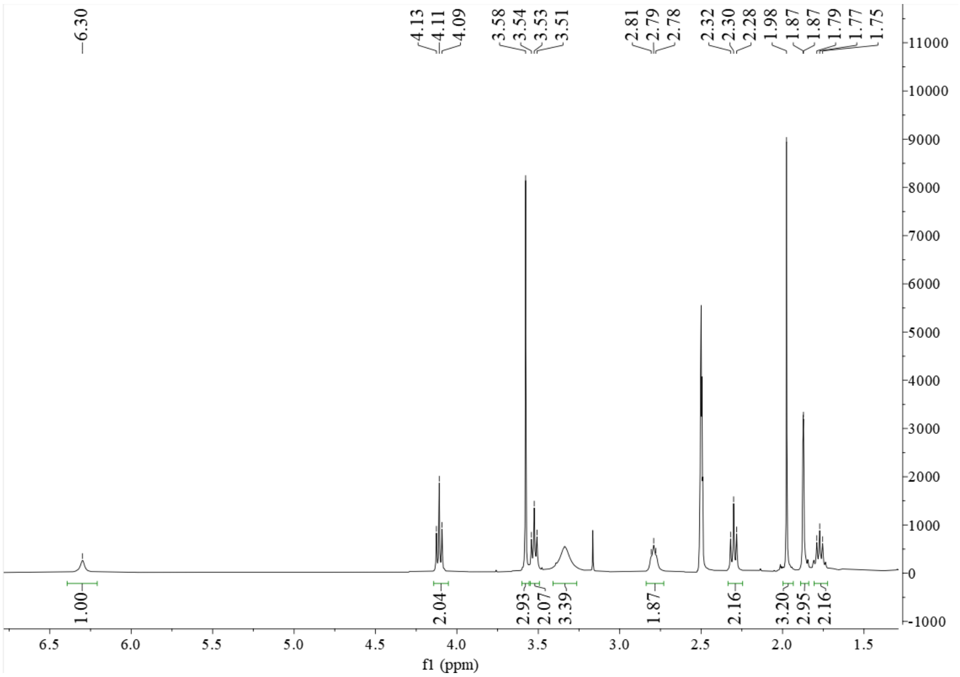

Figure S26. ^{1}H NMR spectrum of **6** in DMSO-d_6

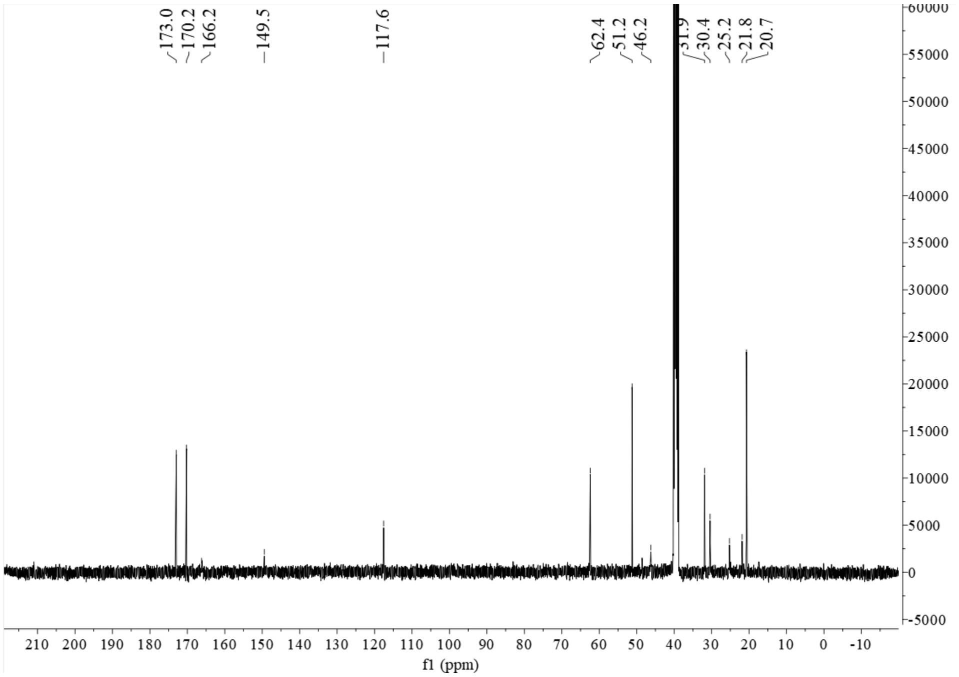

Figure S27. ^{13}C NMR spectrum of **6** in DMSO-d_6

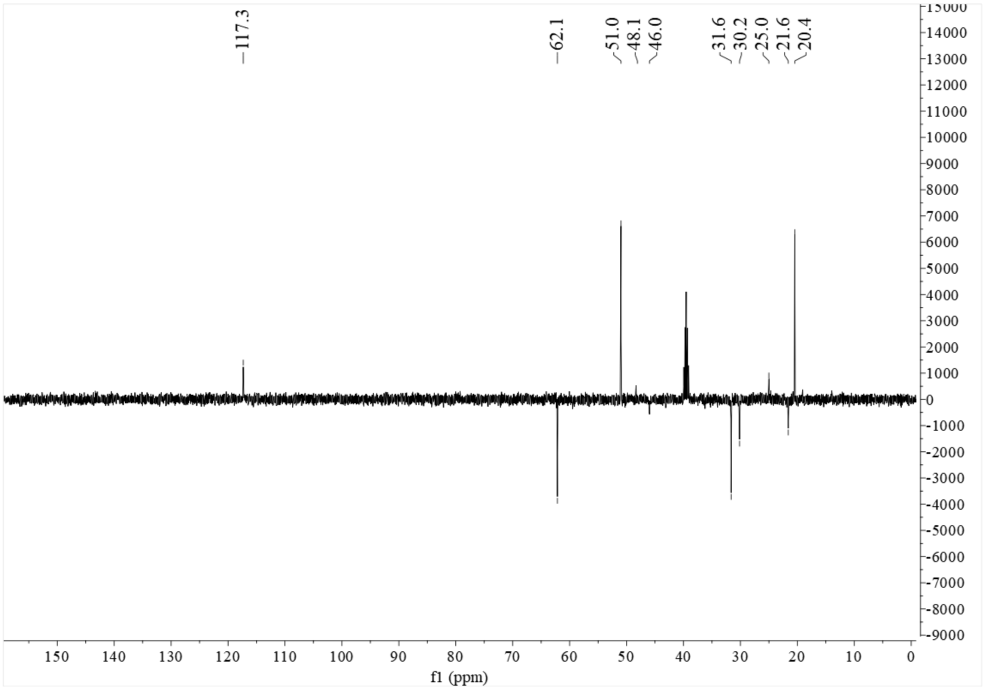

Figure S28. 135-DEPT spectrum of **6** in DMSO-d_6

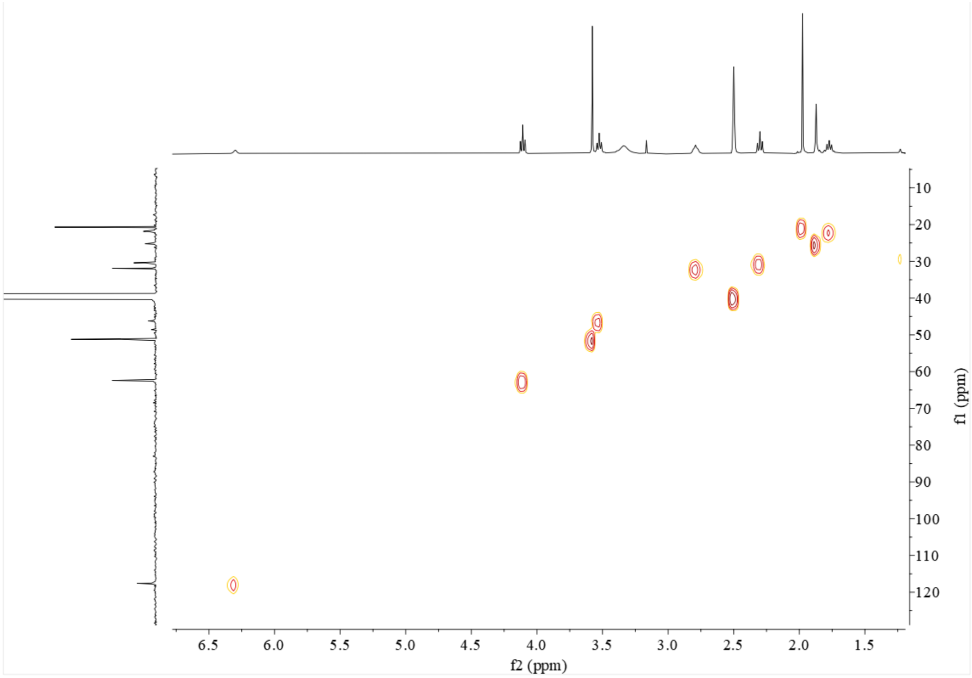

Figure S29. HSQC spectrum of **6** in DMSO-d_6

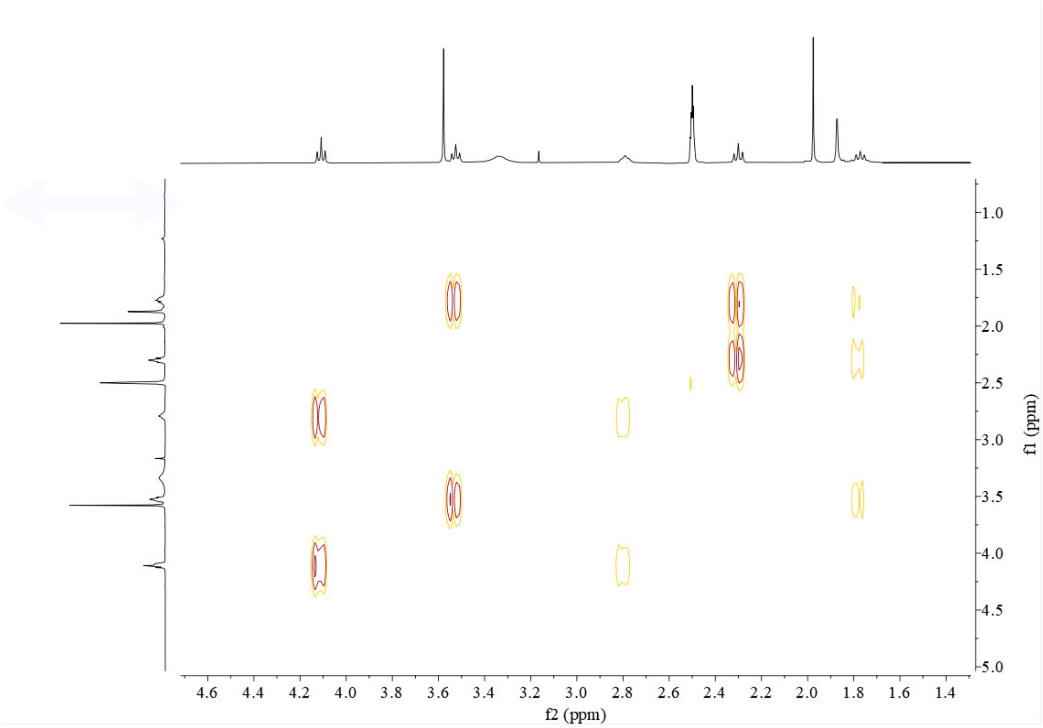

Figure S30. ^1H-^1H COSY spectrum of **6** in DMSO-d_6

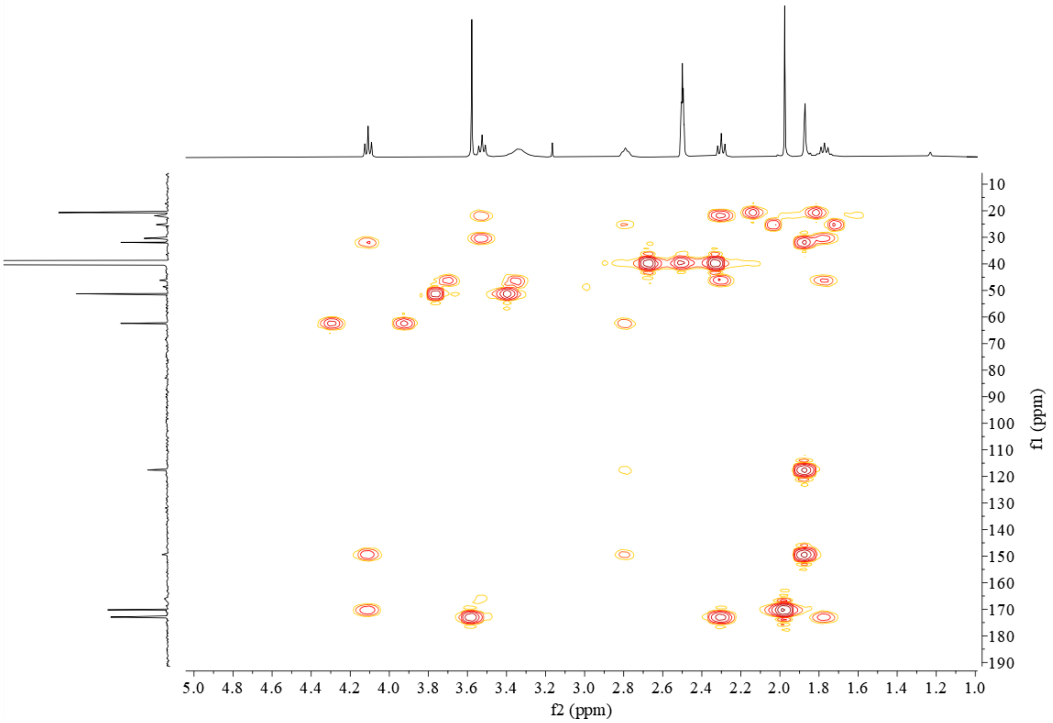

Figure S31. HMBC spectrum of **6** in DMSO-d_6

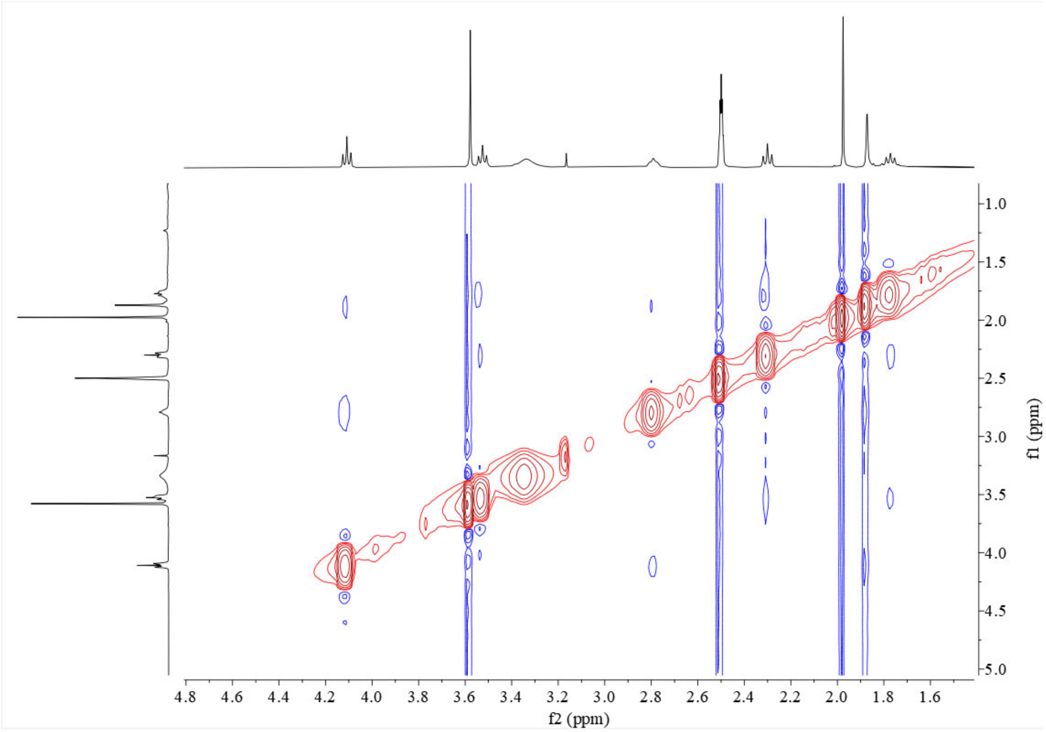

Figure S32. NOESY spectrum of **6** in DMSO-*d*6

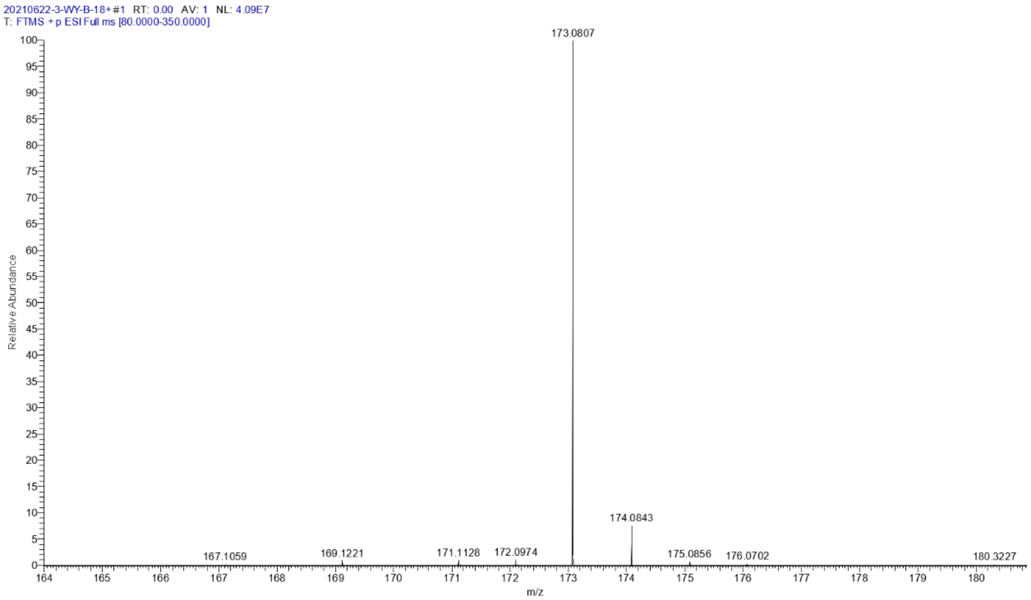

Figure S33. HR-ESI-MS spectrum of **7**

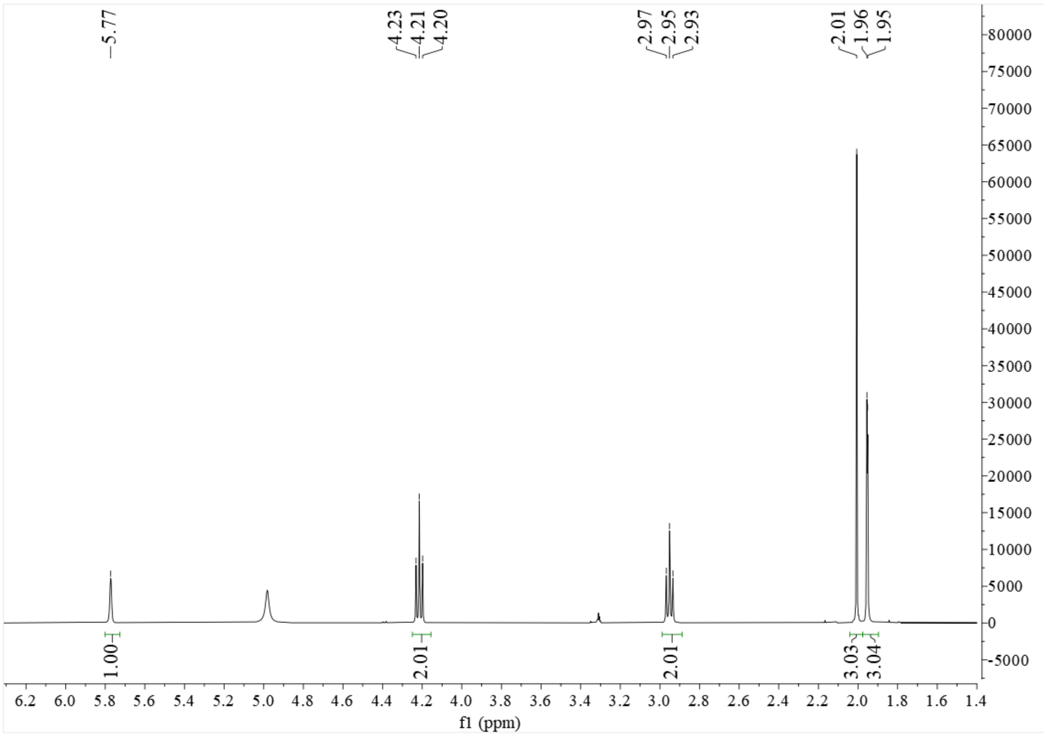

Figure S34. ^{1}H NMR spectrum of **7** in DMSO-d_6

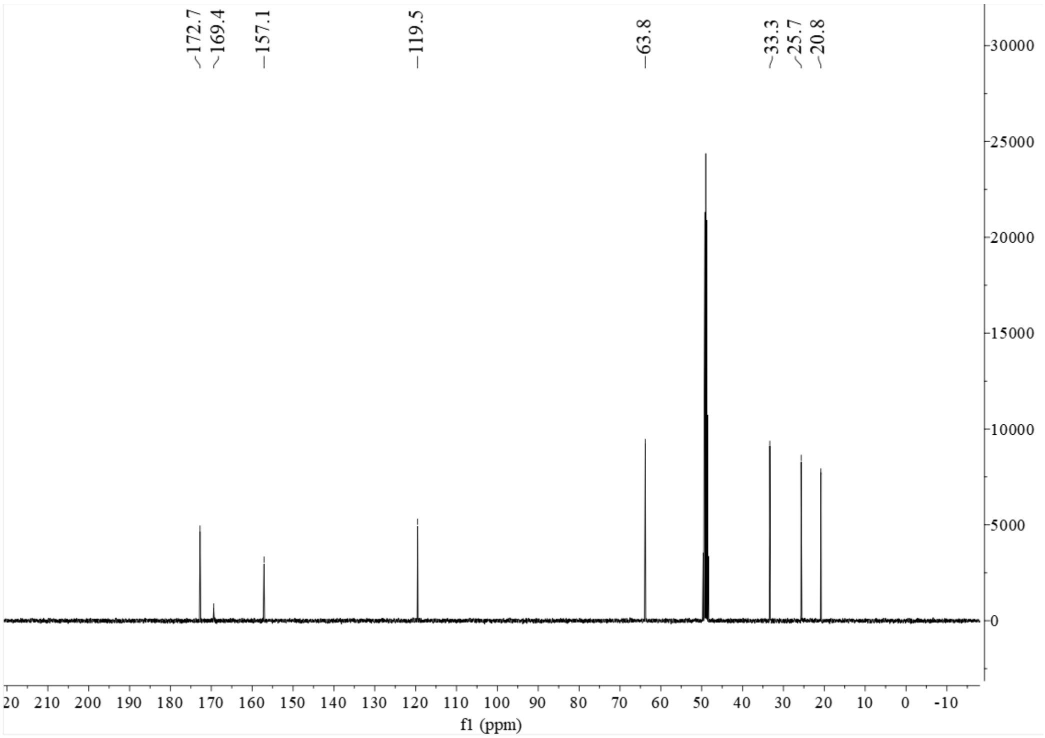

Figure S35. ^{13}C NMR spectrum of **7** in DMSO-d_6

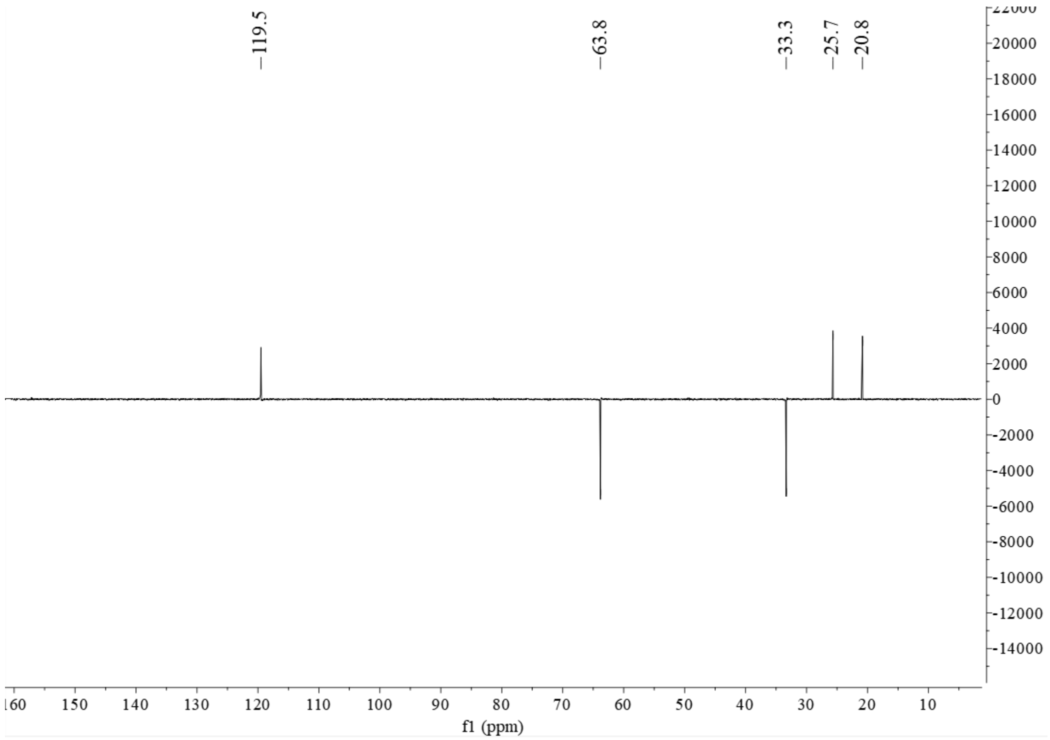

Figure S36. 135-DEPT spectrum of **7** in DMSO-d_6

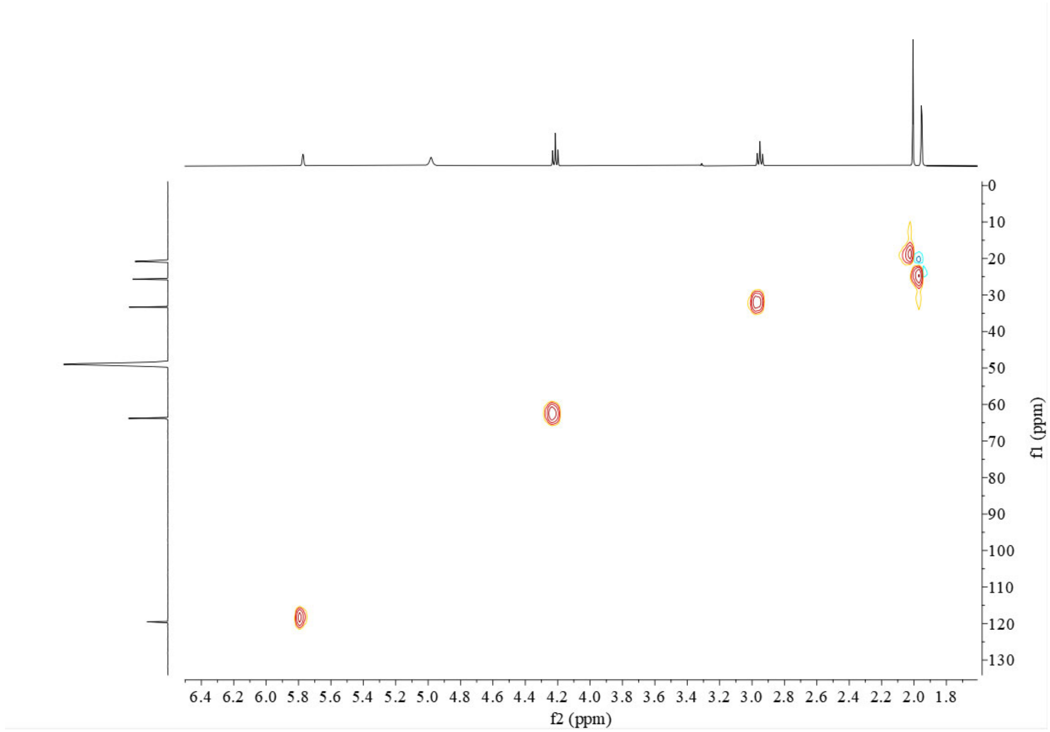

Figure S37. HSQC spectrum of **7** in DMSO-d_6

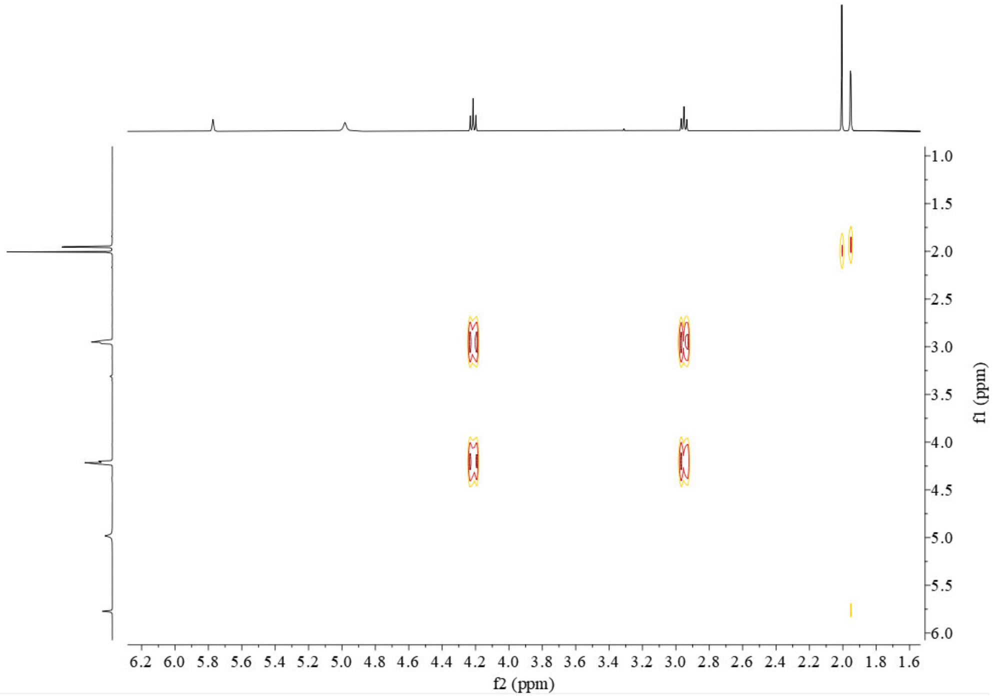

Figure S38. ^{1}H-^{1}H COSY spectrum of **7** in DMSO-d_6

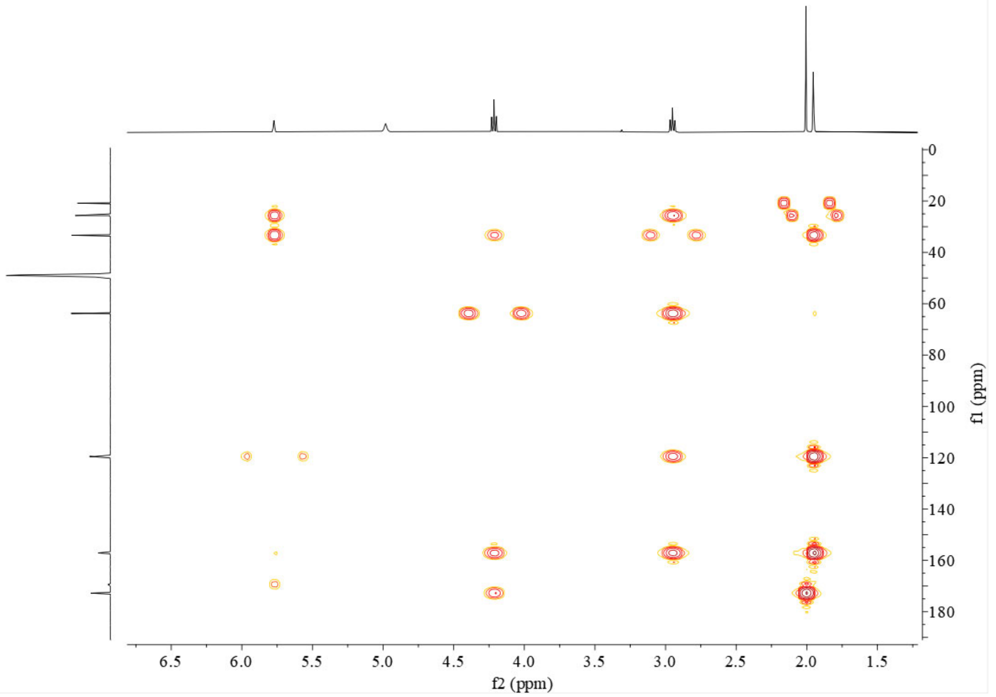

Figure S39. HMBC spectrum of **7** in DMSO-d_6

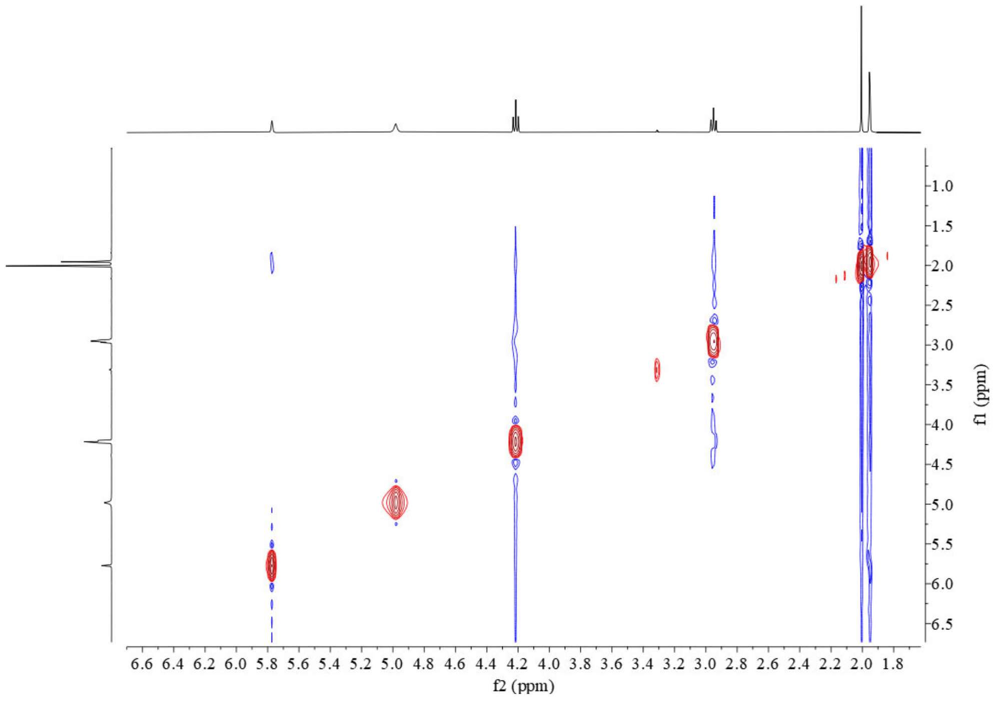

Figure S40. NOESY spectrum of **7** in DMSO-d_6

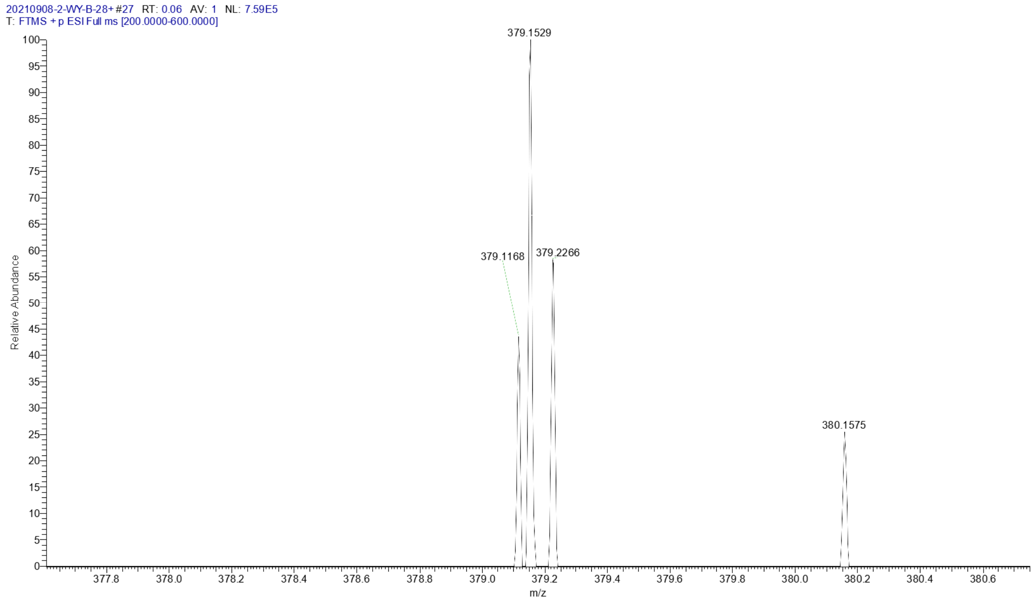

Figure S41. HR-ESI-MS spectrum of **8**

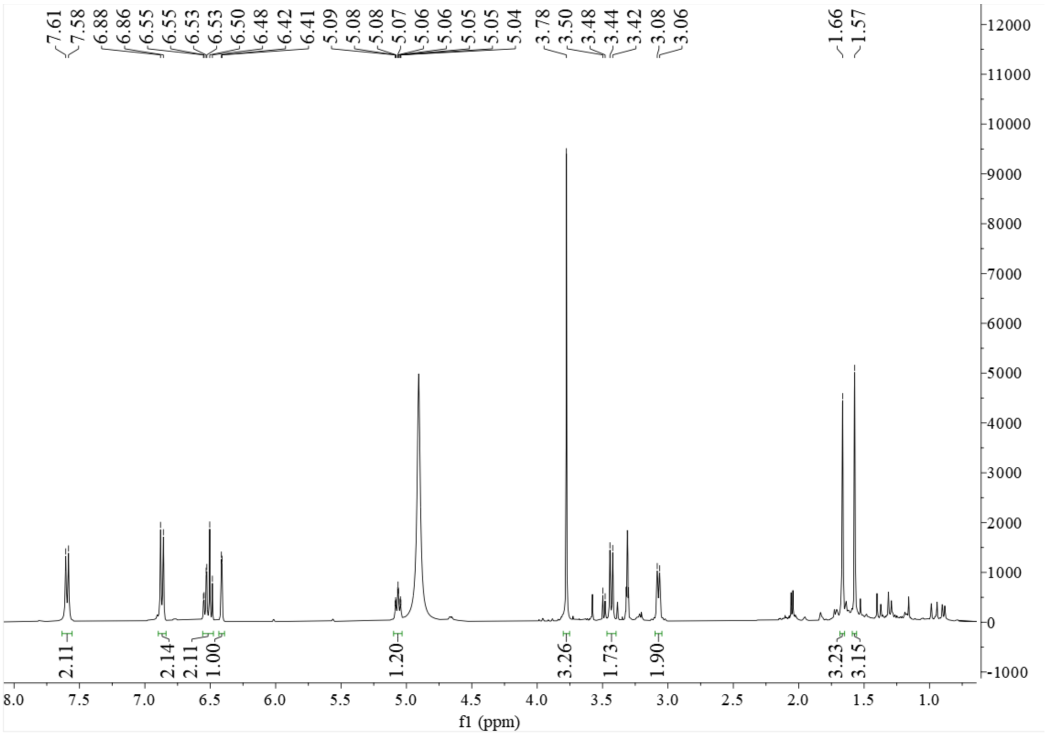

Figure S42. ^{1}H NMR spectrum of **8** in methanol-d_4

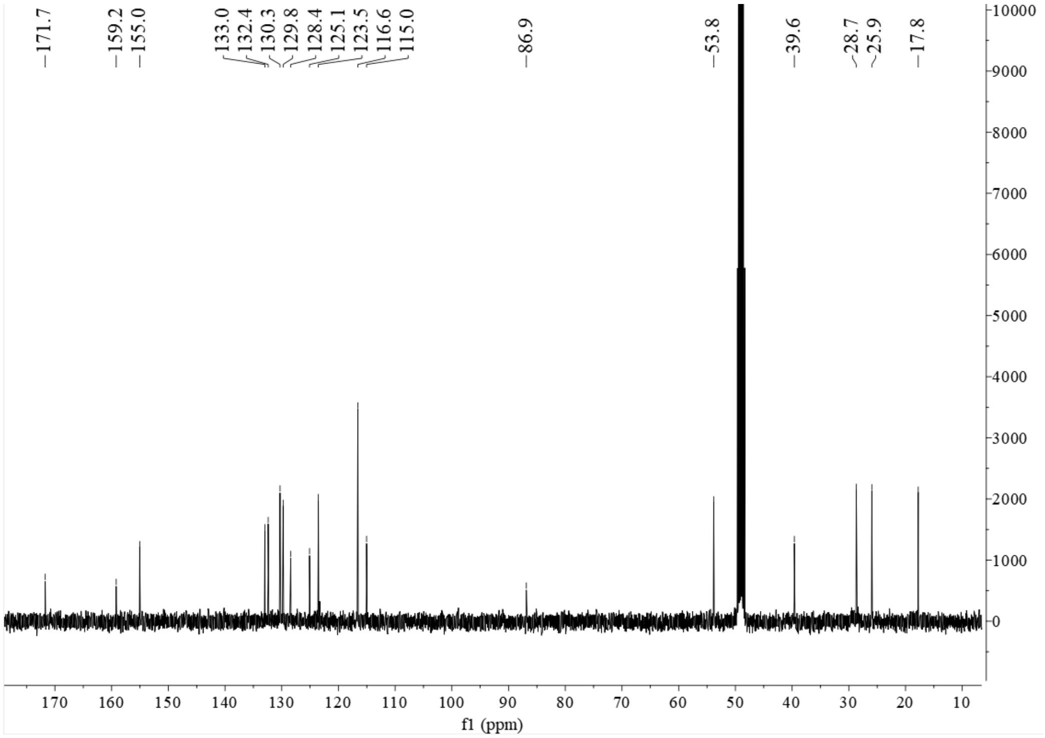

Figure S43. ^{13}C NMR spectrum of **8** in methanol-d_4

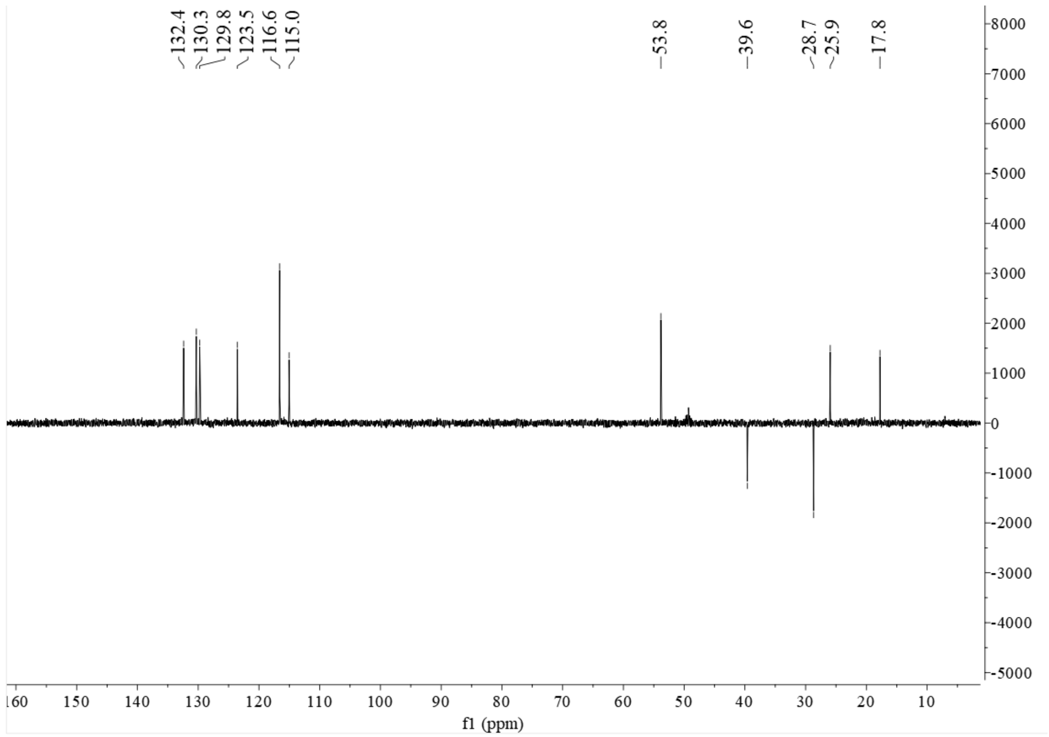

Figure S44. 135-DEPT spectrum of **8** in methanol-d_4

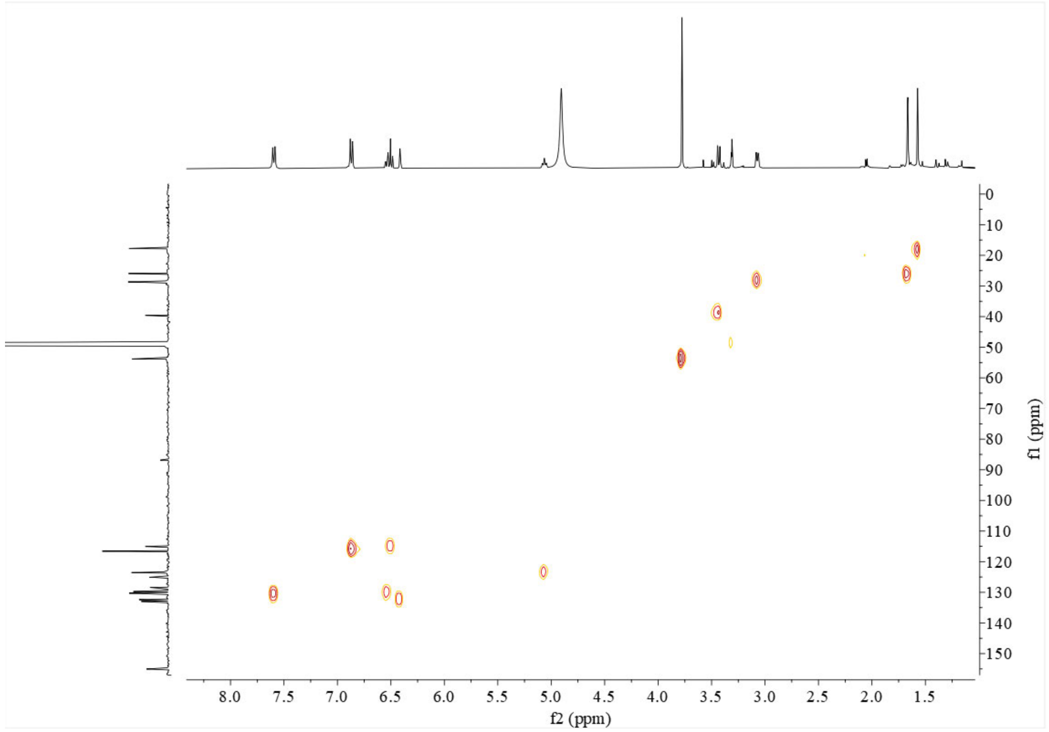

Figure S45. HSQC spectrum of **8** in methanol-d_4

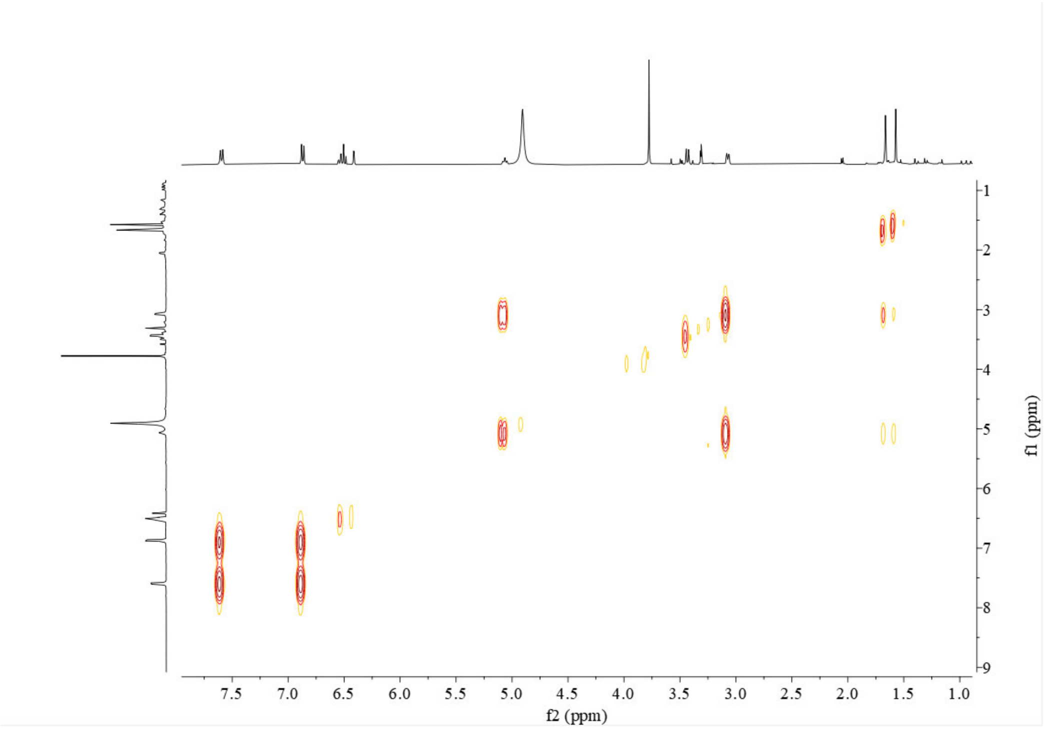

Figure S46. ^1H-^1H COSY spectrum of **8** in methanol-d_4

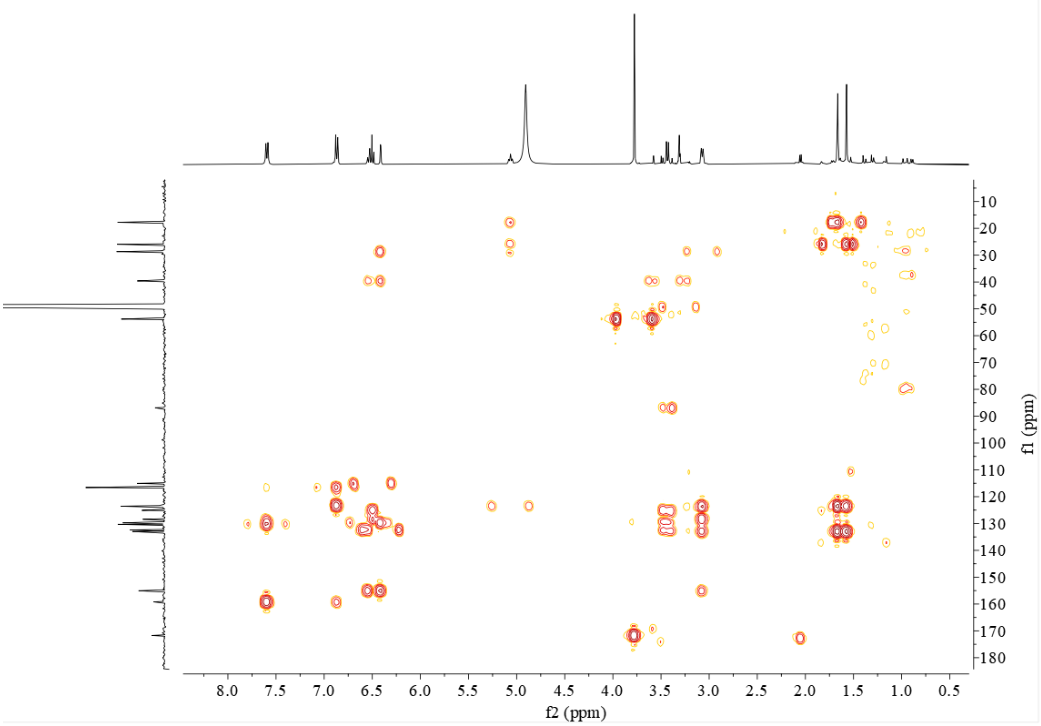

Figure S47. HMBC spectrum of **8** in methanol-d_4

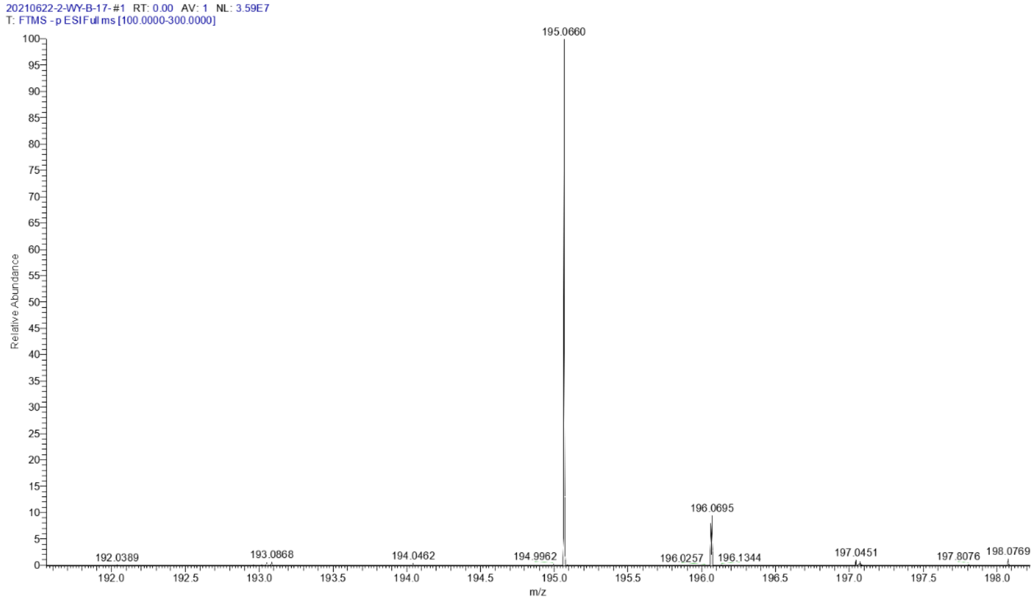

Figure S48. HR-ESI-MS spectrum of **15**

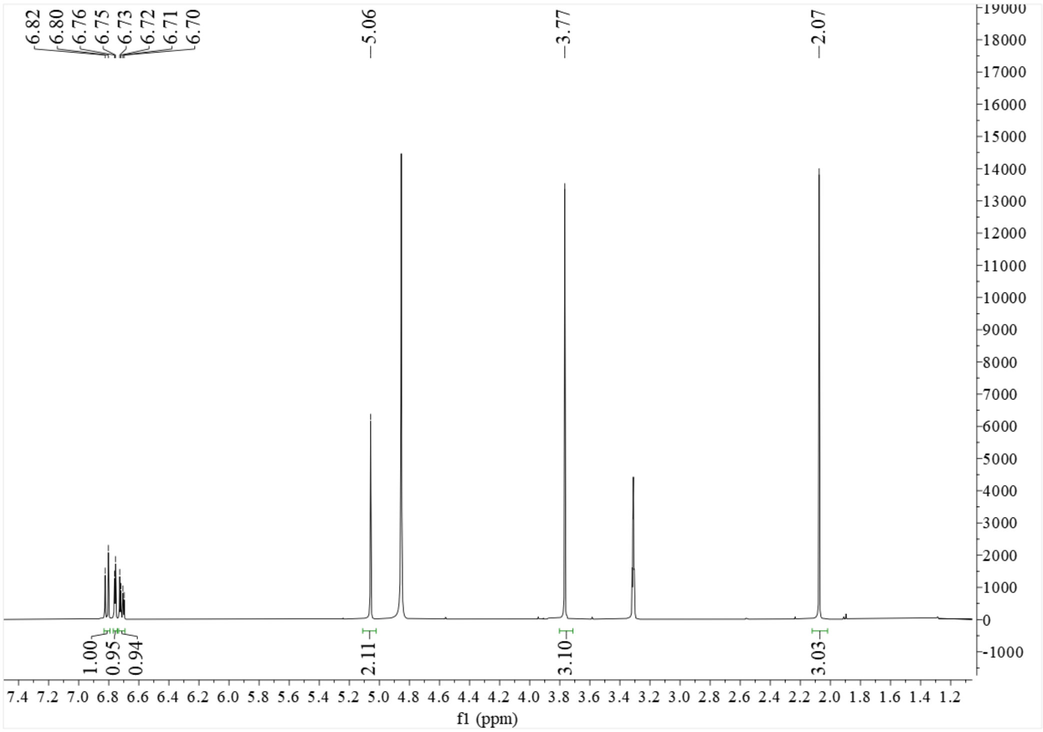

Figure S49. ^{1}H NMR spectrum of **15** in methanol-d_4

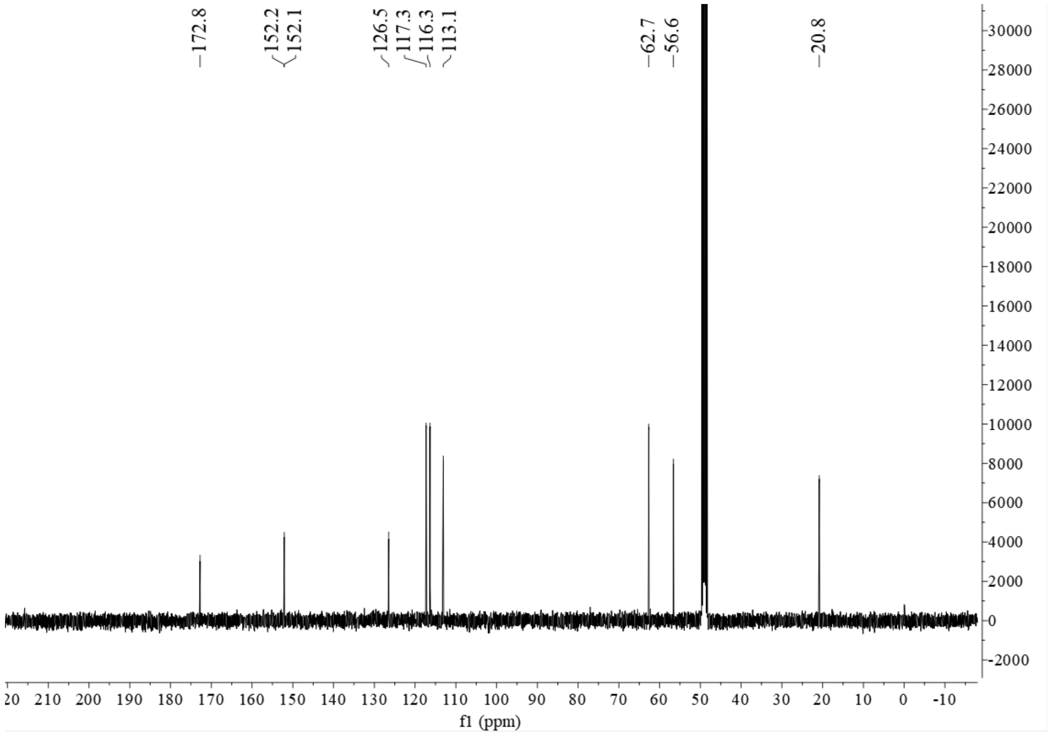

Figure S50. ^{13}C NMR spectrum of **15** in methanol-d_4

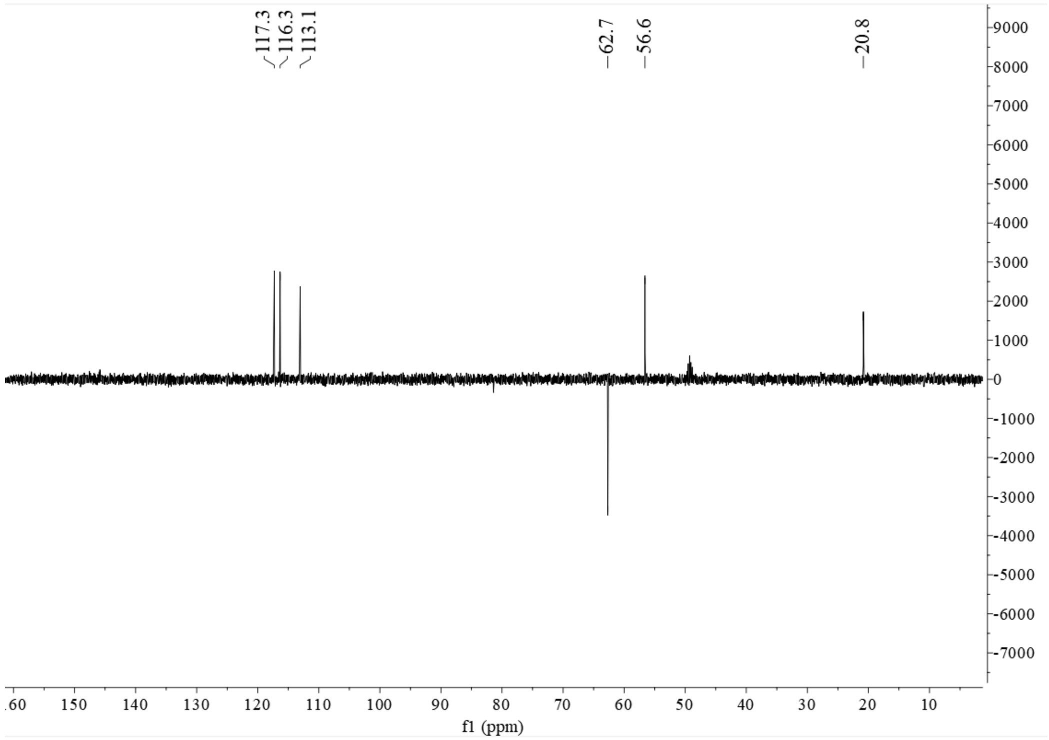

Figure S51. 135-DEPT spectrum of **15** in methanol-d_4

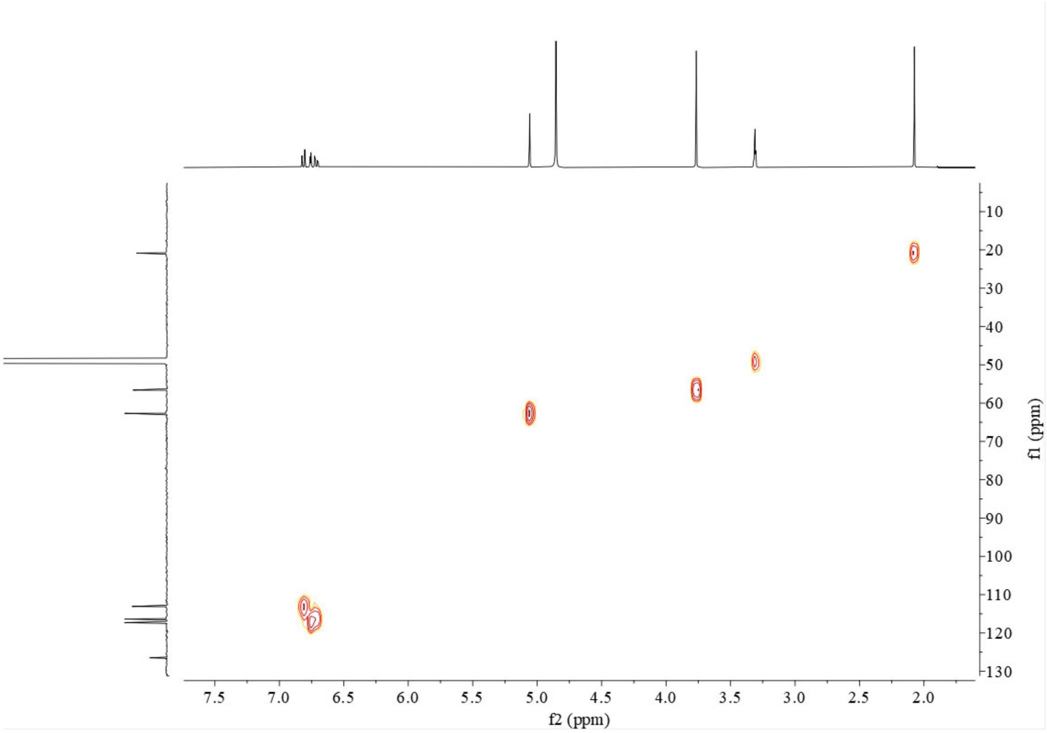

Figure S52. HSQC spectrum of **15** in methanol-*d*$_4$

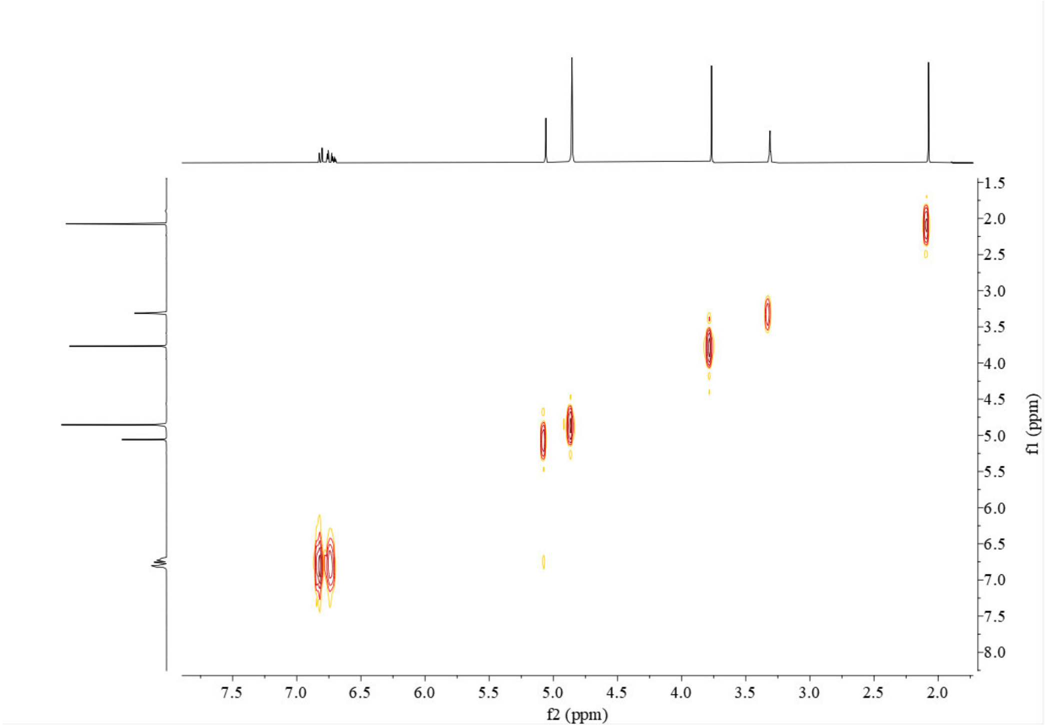

Figure S53. ^{1}H-^{1}H COSY spectrum of **15** in methanol-*d*$_4$

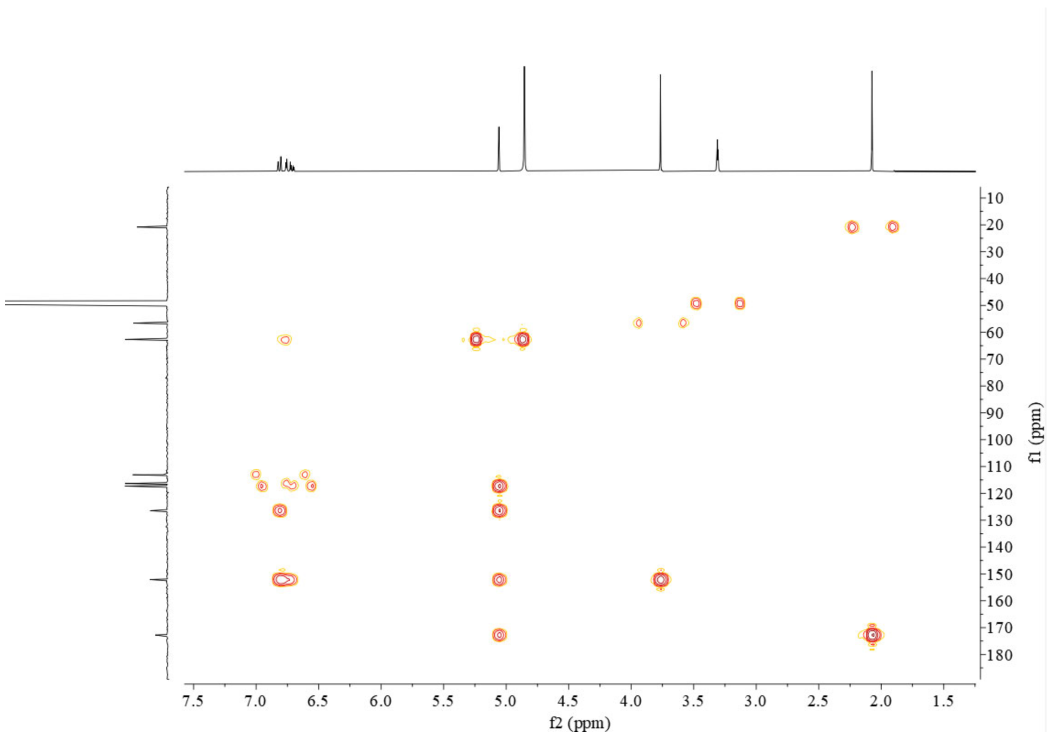

Figure S54. HMBC spectrum of **15** in methanol-d_4

S55: ^{1}H and ^{13}C NMR data of compounds **3-4**, **9-14**

conidiogenone D (**3**): ^{1}H NMR (400 MHz, methanol-d_4) δ_H: 5.95 (1H, dd, *J* = 10.0, 1.1 Hz, H-2), 7.12 (1H, dd, *J* = 10.0, 5.8 Hz, H-3), 2.79 (1H, m, H-4), 2.83 (1H, dd, *J* = 7.6, 5.8 Hz, H-6), 1.59 (1H, m, H-7a), 1.24 (1H, m, H-7b), 2.37(1H, dd, *J* = 9.9, 4.9 Hz, H-8a), 1.70 (1H, m, H-8b), 2.07 (1H, d, *J* = 14.6 Hz, H-10a), 1.71 (1H, d, *J* = 14.6 Hz, H-10b), 1.69 (1H, m, H-12a), 1.55 (1H, m, H-12b), 1.52 (1H, m, H-13a), 1.45 (1H, m, H-13b), 1.50 (1H, d, *J* = 5.8 Hz, H-15), 1.25 (3H, d, *J* = 7.3 Hz, H-16), 1.17 (3H, s, H-17), 1.23 (3H, s, H-18), 3.35 (1H, d, *J* = 9.9 Hz, H-19a), 3.29 (1H, d, *J* = 9.9 Hz, H-19b), 0.98 (3H, s, H-20). ^{13}C NMR (100 MHz, methanol-d_4) δ_C: 208.1 (C-1), 127.8 (C-2), 157.3 (C-3), 39.1 (C-4), 61.0 (C-5), 56.1 (C-6), 35.1 (C-7), 39.7 (C-8), 58.7 (C-9), 47.7 (C-10), 53.9 (C-11), 39.5 (C-12), 37.6 (C-13), 49.3 (C-14), 72.0 (C-15), 18.8 (C-16), 21.6 (C-17), 31.6 (C-18), 69.2 (C-19), 23.1 (C-20).

conidiogenone C (**4**): ^{1}H NMR (400 MHz, methanol-d_4) δ_H: 5.93 (1H, dd, *J* = 10.0, 1.1 Hz, H-2), 7.12 (1H, dd, *J* = 10.0, 5.9 Hz, H-3), 2.75 (1H, m, H-4), 2.39 (1H, dd, *J* = 9.2, 5.4 Hz, H-6), 1.71 (1H, m, H-7a), 1.24 (1H, m, H-7b), 2.02 (1H, ddd, *J* = 11.5, 5.5, 1.7 Hz, H-8a), 1.67 (1H, m, H-8b), 2.06 (1H, d, *J* = 14.6 Hz, H-10a), 1.66 (1H, d, *J* = 14.6 Hz, H-10b), 1.60 (1H, m, H-12a), 1.58 (1H, m, H-12b), 1.55 (1H, m, H-13a), 1.43 (1H, m, H-13b), 1.56 (1H, d, *J* = 5.5 Hz, H-15), 1.22 (3H, d, *J* = 7.3 Hz, H-16), 1.16 (3H, s, H-17), 1.32 (3H, s, H-18), 1.14 (3H, s, H-19), 3.47 (1H, d, *J* = 10.7 Hz, H-20a), 3.37 (1H, d, *J* = 10.7 Hz, H-20b). ^{13}C

NMR (100 MHz, methanol-d_4) δ_C: 208.4 (C-1), 127.9 (C-2), 157.5 (C-3), 39.1 (C-4), 61.2 (C-5), 56.2 (C-6), 35.4 (C-7), 40.0 (C-8), 58.5 (C-9), 48.4 (C-10), 53.5 (C-11), 39.4 (C-12), 37.3 (C-13), 48.9 (C-14), 70.6 (C-15), 19.1 (C-16), 21.4 (C-17), 32.7 (C-18), 26.9 (C-19), 73.0 (C-20).

demethylincisterol A3 (**9**): ^{1}H NMR (400 MHz, methanol-d_4) δ_H: 5.67 (1H, d, *J* = 1.8 Hz, H-2), 2.24 (2H, ddd, *J* = 13.7, 4.0, 2.5 Hz, H-5), 2.67 (1H, ddd, *J* = 12.1, 7.0, 1.7 Hz, H-8), 0.65 (1H, s, H-12), 1.06 (1H, d, *J* = 6.6 Hz, H-14), 5.22 (1H, dd, *J* = 15.3, 7.9 Hz, H-15), 5.29 (1H, dd, *J* = 15.3, 7.2 Hz, H-16), 0.85 (1H, d, *J* = 7.0 Hz, H-19), 0.87 (1H, d, *J* = 6.8 Hz, H-20), 0.95 (1H, d, *J* = 6.8 Hz, H-21). ^{13}C NMR (100 MHz, methanol-d_4) δ_C: 173.6 (C-1), 112.6 (C-2), 173.0 (C-3), 106.7 (C-4), 36.2 (C-5), 36.5 (C-6), 49.9 (C-7), 51.7 (C-8), 22.2 (C-9), 30.1 (C-10), 56.7 (C-11), 12.1 (C-12), 41.5 (C-13), 21.5 (C-14), 136.4 (C-15), 133.8 (C-16), 44.3 (C-17), 34.3 (C-18), 20.4 (C-19), 20.1 (C-20), 18.2 (C-21).

ergosterol (**10**): ^{1}H NMR (400 MHz, $CDCl_3$) δ_H: 3.64 (1H, m, H-3), 2.47 (1H, ddd, *J* = 14.4, 4.7, 2.3 Hz, H-4a), 2.28(1H, t, *J* = 7.6 Hz, H-4b), 5.57 (1H, dd, *J* = 5.6, 2.4 Hz, H-6), 5.38 (1H, m, H-7), 0.63 (3H, s, H-18), 0.94 (3H, s, H-19), 1.03 (3H, d, *J* = 6.6 Hz, H-21), 5.16 (1H, dd, *J* = 15.3, 7.5 Hz, H-22), 5.24 (1H, dd, *J* = 15.3, 7.5 Hz, H-23), 0.82 (3H, d, *J* = 6.4 Hz, H-26), 0.84 (3H, d, *J* = 6.4 Hz, H-27), 0.92 (3H, d, *J* = 6.8 Hz, H-28). ^{13}C NMR (100 MHz, $CDCl_3$) δ_C: 38.5 (C-1), 32.2 (C-2), 70.6 (C-3), 41.0 (C-4), 139.9 (C-5), 119.7 (C-6), 116.4 (C-7), 141.5 (C-8),

46.4 (C-9), 37.2 (C-10), 21.3 (C-11), 39.2 (C-12), 43.0 (C-13), 54.7 (C-14), 23.2 (C-15), 28.4 (C-16), 55.9 (C-17), 12.2 (C-18), 16.4 (C-19), 40.6 (C-20), 21.3 (C-21), 135.7 (C-22), 132.1 (C-23), 43.0 (C-24), 33.2 (C-25), 19.8 (C-26), 20.1 (C-27), 17.8 (C-28).

Δ[7]-sitosterol (**11**): ^{1}H NMR (400 MHz, $CDCl_3$) δ_H: 3.65 (1H, m, H-3), 2.48 (1H, d, *J* = 12.0 Hz, H-4a), 2.29(1H, t, *J* = 12.0 Hz, H-4b), 5.58 (1H, d, *J* = 5.7 Hz, H-6), 5.40 (1H, d, *J* = 5.7 Hz, H-7), 0.63 (3H, s, H-18), 0.96 (3H, s, H-19), 0.97 (3H, d, *J* = 6.0 Hz, H-21), 0.80 (3H, d, *J* = 6.6 Hz, H-26), 0.83 (3H, d, *J* = 6.6 Hz, H-27), 0.85 (3H, d, *J* = 6.6 Hz, H-28). ^{13}C NMR (100 MHz, $CDCl_3$) δ_C: 38.3 (C-1), 32.1 (C-2), 72.6 (C-3), 40.6 (C-4), 139.9 (C-5), 120.3 (C-6), 116.5 (C-7), 141.6 (C-8), 46.2 (C-9), 37.2 (C-10), 20.1 (C-11), 39.2 (C-12), 43.0 (C-13), 54.7 (C-14), 22.8 (C-15), 28.3 (C-16), 55.9, (C-17), 12.2 (C-18), 16.3 (C-19), 36.8 (C-20), 19.8 (C-21), 36.3 (C-22), 25.2 (C-23), 146.9 (C-24), 34.9 (C-25), 21.3 (C-26), 21.2 (C-27), 115.6 (C-28), 14.3 (C-28).

(-)-*β*-sitosterol (**12**): ^{1}H NMR (400 MHz, $CDCl_3$) δ_H: 3.52 (1H, tq, *J* = 9.5, 4.4 Hz, H-3a), 5.35 (1H, d, *J* = 5.4 Hz, H-6), 0.68 (3H, s, H-18), 1.01 (3H, s, H-19), 0.92 (3H, d, *J* = 6.6 Hz, H-21), 0.83 (3H, d, *J* = 7.4 Hz, H-26), 0.81 (3H, d, *J* = 6.9 Hz, H-27), 0.85 (3H, d, *J* = 7.5 Hz, H-29). ^{13}C NMR (100 MHz, $CDCl_3$) δ_C: 37.4 (C-1), 31.8 (C-2), 72.0 (C-3), 42.5 (C-4), 140.9 (C-5), 121.9 (C-6), 32.1 (C-7), 56.9 (C-8), 36.7 (C-9), 21.2 (C-11), 39.9 (C-12), 46.0 (C-13), 56.2 (C-14), 24.5 (C-15), 28.4

(C-16), 26.3 (C-17), 12.1 (C-18), 20.0 (C-19), 36.3 (C-20), 18.9 (C-21), 34.1 (C-22), 26.3 (C-23), 50.3 (C-24), 29.3 (C-25), 19.5 (C-26), 19.2 (C-27), 23.2 (C-28), 12.0 (C-29).

7-deacetoxyyanuthone A (**13**): ^{1}H NMR (400 MHz, methanol-d_4) δ_H: 3.64 (1H, d, *J* = 3.0 Hz, H-2), 4.41 (1H, s, H-4), 5.77 (1H, s, H-5), 1.97 (3H, s, H-7), 2.38 (1H, dd, *J* = 15.5, 7.0 Hz, H-8a), 2.75 (1H, dd, *J* = 15.5, 7.0 Hz, H-8b), 5.01 (1H, t, *J* = 7.0 Hz, H-9), 2.03 (2H, m, H-11), 2.10 (2H, m, H-12), 5.07 (1H, t, *J* = 7.0 Hz, H-13), 2.06 (2H, m, H-15), 2.08 (2H, m, H-16), 5.09 (1H, t, *J* = 7.0 Hz, H-17), 1.66 (3H, s, H-19), 1.59 (3H, s, H-20), 1.59 (3H, s, H-21), 1.65 (3H, s, H-22). ^{13}C NMR (100 MHz, methanol-d_4) δ_C: 195.6 (C-1), 60.5 (C-2), 68.1 (C-3), 61.6 (C-4), 159.2 (C-5), 125.1 (C-6), 20.3 (C-7), 27.7 (C-8), 118.1 (C-9), 140.0 (C-10), 40.8 (C-11), 27.3 (C-12), 123.6 (C-13), 136.0 (C-14), 40.8 (C-15), 27.4 (C-16), 125.4 (C-17), 131.9 (C-18), 26.0 (C-19), 17.9 (C-20), 16.2 (C-21), 16.5 (C-22).

(1*S*,5*R*,6*S*)-5-Hydroxy-4-methyl-1-[(2*E*,6*E*)-3,7,11-trimethyl-2,6,10-dodecatrien-1-yl]-7-oxabicyclo[4.1.0]hept-3-en-2-one (**14**): ^{1}H NMR (400 MHz, methanol-d_4) δ_H: 4.15 (1H, d, *J* = 3.4 Hz, H-2), 4.70 (1H, s, H-4), 5.83 (1H, s, H-5), 1.99 (3H, s, H-7), 2.72 (1H, dd, *J* = 15.5, 7.3 Hz, H-8a), 2.81 (1H, dd, *J* = 15.5, 7.3 Hz, H-8b), 5.28 (1H, t, *J* = 7.0 Hz, H-9), 2.07 (2H, m, H-11), 2.27 (2H, m, H-12), 5.32 (1H, t, *J* = 7.0 Hz, H-13), 2.12 (2H, m, H-15), 2.13 (2H, m, H-16), 5.35 (1H, t, *J* = 7.0 Hz, H-17), 1.69 (3H, s, H-19), 1.62 (3H, s, H-20), 1.60 (3H, s, H-21), 1.67 (3H, s,

H-22). ^{13}C NMR (100 MHz, methanol-d_4) δ_C: 193.3 (C-1), 60.0 (C-2), 75.3 (C-3), 71.2 (C-4), 162.4 (C-5), 125.3 (C-6), 20.5 (C-7), 32.2 (C-8), 118.2 (C-9), 140.9 (C-10), 41.2 (C-11), 27.5 (C-12), 124.0 (C-13), 136.0 (C-14), 40.9 (C-15), 27.8 (C-16), 125.5 (C-17), 132.1 (C-18), 25.9 (C-19), 17.8 (C-20), 16.2 (C-21), 16.8 (C-22).
